# Prevalence and Impact of Biofilms on Bloodstream and Urinary Tract Infections: A Systematic Review and Meta-Analysis

**DOI:** 10.3390/antibiotics10070825

**Published:** 2021-07-08

**Authors:** Henrique Pinto, Manuel Simões, Anabela Borges

**Affiliations:** 1 LEPABE—Laboratory for Process Engineering, Environment, Biotechnology and Energy, Faculty of Engineering, University of Porto, Rua Dr. Roberto Frias, s/n, 4200-465 Porto, Portugal; up201708963@fe.up.pt (H.P.); mvs@fe.up.pt (M.S.); 2 DEQ—Department of Chemical Engineering, Faculty of Engineering, University of Porto, Rua Dr. Roberto Frias, s/n, 4200-465 Porto, Portugal

**Keywords:** biofilms, bloodstream infections, clinical outcomes, multi-drug resistant bacteria, urinary tract infections

## Abstract

This study sought to assess the prevalence and impact of biofilms on two commonly biofilm-related infections, bloodstream and urinary tract infections (BSI and UTI). Separated systematic reviews and meta-analyses of observational studies were carried out in PubMed and Web of Sciences databases from January 2005 to May 2020, following PRISMA protocols. Studies were selected according to specific and defined inclusion/exclusion criteria. The obtained outcomes were grouped into biofilm production (BFP) prevalence, BFP in resistant vs. susceptible strains, persistent vs. non-persistent BSI, survivor vs. non-survivor patients with BSI, and catheter-associated UTI (CAUTI) vs. non-CAUTI. Single-arm and two-arm analyses were conducted for data analysis. In vitro BFP in BSI was highly related to resistant strains (odds ratio-OR: 2.68; 95% confidence intervals-CI: 1.60–4.47; *p* < 0.01), especially for methicillin-resistant *Staphylococci*. BFP was also highly linked to BSI persistence (OR: 2.65; 95% CI: 1.28–5.48; *p* < 0.01) and even to mortality (OR: 2.05; 95% CI: 1.53–2.74; *p* < 0.01). *Candida* spp. was the microorganism group where the highest associations were observed. Biofilms seem to impact *Candida* BSI independently from clinical differences, including treatment interventions. Regarding UTI, multi-drug resistant and extended-spectrum *β*-lactamase-producing strains of *Escherichia coli*, were linked to a great BFP prevalence (OR: 2.92; 95% CI: 1.30–6.54; *p* < 0.01 and OR: 2.80; 95% CI: 1.33–5.86; *p* < 0.01). More in vitro BFP was shown in CAUTI compared to non-CAUTI, but with less statistical confidence (OR: 2.61; 95% CI: 0.67–10.17; *p* < 0.17). This study highlights that biofilms must be recognized as a BSI and UTI resistance factor as well as a BSI virulence factor.

## 1. Introduction

Biofilms are the predominant lifestyle microorganisms and an example of a successful physiological adaptation, as they thrive in most natural environments as well as in harsh conditions [1]. They are also often associated with many pathogenic forms of human diseases and can negatively impact health [1]. Indeed, biofilms are typically pathogenic and can cause serious infections, as they offer to microorganisms an enormous capacity to resist host immune system defenses and antimicrobial therapy [2]. In healthcare environments, the persistence of the microorganisms is extended by the formation of biofilms, being responsible for the onset and spread of hospital-care-associated infections (HCAIs) (also referred to as “nosocomial” or “hospital” infections) [3]. HCAIs can result in prolonged hospital stays, long-term disabilities and overcrowded communities of patients treated together, enhanced resistance of the microorganisms to antimicrobials, enormous additional costs for health care systems, high costs for patients and their families, and unnecessary deaths. The prevalence of HCAI is estimated to be between 5.7% and 19.1%. In the United States (US), it is estimated that the mortality rate due to HCAI is around 5.8% [4]. Furthermore, growing evidence indicates that chronic or persistent bacterial infections are greatly linked to biofilm formation, differing from the planktonic bacteria found in acute infections, which are in general more easily treated [5].

Regardless, planktonic antimicrobial susceptibility testing is often performed by clinics to assess the treatment choice for biofilm-associated infections (BAIs), resulting in underestimated antibiotic/antifungal doses [6,7]. Another problem related to clinical microbiology laboratories is that, even though having limitations, culture and microscopy are still two of the most utilized techniques [8]. These methods do not identify most bacteria/fungi in the complex polymicrobial communities such as those found in BAIs [9,10]. For instance, the conventional culturing methods only identify circa 1% of the bacteria in a chronic wound [11]. Molecular techniques can overcome this challenge; however, their implementation is not widespread due to higher costs and the degree of know-how required [12]. Thus, these clinical diagnostic and assessment tests can fail, leading to unsuccessful infection treatments and constant re-infection. Consequently, substantial healthcare costs and some selective pressure on microorganisms that can further lead to increased antimicrobial resistance are imposed [7,12,13,14].

According to some estimates, 65–80% of total human infections are associated with biofilm formation and include: periodontitis/dental caries, cystic fibrosis lung infection, chronic otitis media, infective endocarditis, chronic osteomyelitis, chronic rhinosinusitis, chronic tonsillitis, chronic peritonitis, chronic prostatitis, chronic wounds, recurrent urinary tract infections (UTIs), bloodstream infections (BSIs), ventilated-associated pneumonia and infections associated with indwelling medical devices (e.g., contact lenses, heart valves, joint prostheses, and other orthopedic implants, intrauterine devices, intravascular catheters, urinary tract catheters, peritoneal catheters, etc.) [2,5,14,15,16,17,18,19].

From the mentioned infections, UTIs are amongst the most common bacterial infection in humans, representing a severe public health issue. The societal costs of these infections, including healthcare costs, are around US$3.5 billion per year in the US [20]. UTIs result in many types of complications such as cystitis, pyelonephritis, prostatitis, urethritis, and bacteriuria [21,22,23]. Urinary catheters provide the ideal environment for the attachment and subsequent colonization by uropathogens [20,23]. Large parts of the biofilms or high concentrations of microbial cells can detach from the catheter and enter into the bladder leading to bacteriuria [24]. Besides, uropathogens can form biofilms in the bladder and kidney, reducing antibiotic susceptibility and ultimately causing infection relapse or recurrence. Therefore, biofilms play a central role in catheter-associated UTIs (CAUTIs) and at some point for prolonged catheterization. CAUTIs are related to increased morbidity and mortality [23]. The most common agents responsible for complicated UTIs (from the most prevalent to less prevalent) are uropathogenic *Escherichia coli*, *Enterococcus* spp., *Klebsiella pneumoniae*, *Candida* spp., *Staphylococcus aureus*, *Proteus mirabilis* and *Pseudomonas aeruginosa*. It is important to highlight that all these species have already been linked to biofilm formation [20].

Not less relevant and worrying than UTIs are the BSIs where the estimated overall mortality rate is 15–30%. In 2017, these infections were ranked as the 12th leading cause of death (associated with septicemia) in the US [25,26,27]. The development of BSIs is related to the presence of bacteria (bacteremia) or fungus (fungemia) in the blood that is normally a sterile environment [28]. Bacteria and fungus can travel through the bloodstream to distant sites in the body, causing hematogenous spread. Thus, BSIs can cause or be caused by localized infections such as endocarditis, pneumonia, UTI, meningitis, osteomyelitis, prosthetic infections, and others [28,29]. Besides, catheter-related BSIs are linked with the presence of bacteremia/fungemia originated from an intravenous catheter. It is the most common cause of nosocomial bacteremia and the main complication associated with catheterization [30].

Resistance to antibiotic therapy due to biofilm formation has an important role in BSIs development. Since biofilms are often present in most of the above-mentioned infections but mainly because they can easily form after catheter insertion, it represents a risk factor to patients [30]. In fact, when biofilm development occurs on the intravenous/intravascular catheter, the propensity to treatment failure increases and so precludes the establishment of chronic infections is almost impossible [27,28]. Furthermore, biofilm dispersion will inevitably happen, releasing high concentrations of bacteria/fungus into the bloodstream [31,32]. In a time span of 20 years (1997–2016), the ten main bacteria recorded as causative BSIs were *S. aureus*, *E. coli*, *K. pneuomoniae*, *P. aeruginosa*, *Enterococcus faecalis*, *Staphylococcus epidermidis*, *Enterobacter cloacae*, *Streptococcus pneumoniae*, *Enterococus faecium* and *Acinetobacter baumannii* [33]. On the other hand, one also has Candidemia, which, although less frequent, is the major cause of patient morbidity and mortality. Note that its incidence has increased in the past two decades, being more than 90% of candidemia cases caused by *Candida albicans*, *Candida glabrata*, *Candida parapsilosis*, *Candida tropicalis*, and *Candida krusei* species. All the mentioned microbial species have been highly associated with biofilm formation [34,35].

The aim of this study was to provide a comprehensive analysis of the literature on biofilm prevalence and its impact on resistance and clinical outcomes of BSIs and UTIs. This was accomplished by carrying out a systematic review and meta-analysis in order to provide insightful data and clarifications.

## 2. Results and Discussion

### 2.1. Bloodstream Infections

#### 2.1.1. Literature Search and Study Selection

**Figure 1:**
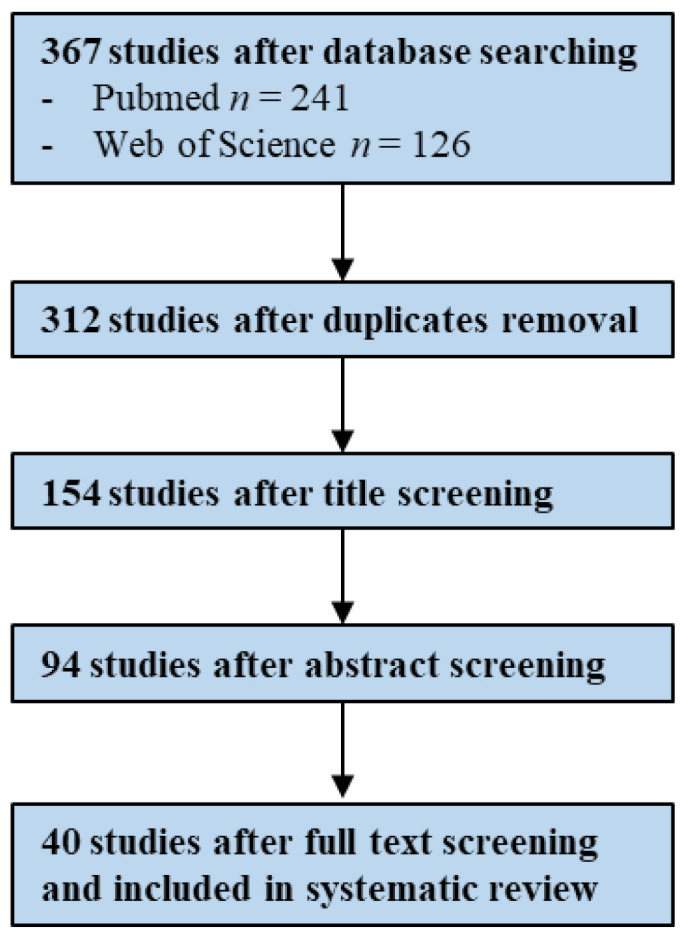
Flowchart illustrating the study screening process—BSIs (Bloodstream Infections).

The systematic on PubMed and Web of Science databases generated a total of 367 studies of which 40 were identified as eligible after duplicates removal, title, abstract and full-text screening based on inclusion and exclusion criteria (Figure 1).

#### 2.1.2. Study Characteristics

Of the 40 eligible studies, biofilm production (BFP) unrelated prevalence data were retrieved from 28 studies, six studies for BFP prevalence related to resistance, five to persistence and 10 to mortality. Only one study had data eligible for all analysis, another single study for BFP unrelated prevalence, related to resistance and mortality, two studies for prevalence and mortality and five studies shared data for prevalence and resistance (Supplementary Materials; S2. Study characteristics; Tables S1–S4).

*Candida* spp. (*n* = 5), *C. parapsilosis* (*n* = 1), *Corynebacterium* spp. (*n* = 1), *E. coli* (*n* = 4), *E. faecalis* (*n* = 1), *Staphylococcus* spp. (*n* = 4), *S. aureus* (*n* = 4), *S. epidermidis* (*n* = 5), *Staphylococcus haemolyticus* (*n* = 2) and *Streptococcus* spp. (*n* = 1) were the pathogens reported in BFP unrelated prevalence analysis. For prevalence related to resistance, the reported pathogens were: *Staphylococcus* spp. (*n* = 1) *S. aureus* (*n* = 3), *S. epidermidis* (*n* = 1) and *E. coli* (*n* = 1). For BFP prevalence related to persistence: *Candida* spp. (*n* = 3), Staphylococcus spp. (*n* = 1) and *S. aureus* (*n* = 1). Finally, for prevalence related to mortality: *Candida* spp. (*n* = 5), *C. parapsilosis* (*n* = 1), *Chryseobacterium meningosepticum* (*n* = 1), *E. coli* (*n* = 2) and *S. aureus* (*n* = 1) (Supplementary Materials; S2. Study characteristics; Tables S1–S4).

#### 2.1.3. BFP Unrelated Prevalence: Single-Armed Meta-Analysis

**Figure 2:**
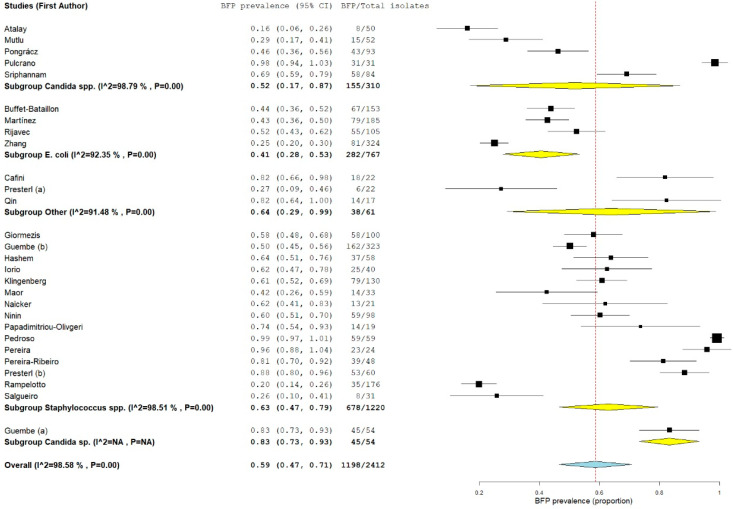
Forest plot of BFP (biofilm production) prevalence and subgroup analysis by microorganism “P” represents the *p*-value. The “I^2^” statistic describes the percentage of variation across studies that is due to heterogeneity rather than chance, where “CI” stands for confidence interval.

Combined results from all 28 studies were pooled in a forest plot (Figure 2) (proportion 0.59; 95% confidence interval-CI: 0.47–0.71; *p* < 0.01). Sub-groups were presented by group of microorganism. The highest proportion was estimated for the other microorganisms “subgroup other” (proportion 0.64; 95% CI: 0.29–0.99; *p* < 0.01), followed by “*Staphylococcus* spp. subgroup” (proportion 0.63; 95% CI: 0.47–0.79; *p* < 0.01).

The estimate for the “*Candida* spp. subgroup” was not much lower (proportion 0.57; 95% CI: 0.28–0.86; *p* < 0.01). The lowest estimate proportion was found for the “*E. coli* subgroup” (proportion 0.41; 95% CI: 0.28–0.53; *p* < 0.01). Despite the BFP proportion being considerable for all subgroups, estimates and 95% CIs varied deeply, which resulted in very high heterogeneities (all subgroups with I^2^ > 90% and *p* < 0.01) and made the meta-analysis unreliable and consequently inconclusive. Since a single-arm analysis does not include a comparison or control group, specific criteria were included for the BFP method to attempt to diminish heterogeneity (see Section 2.2). There are many other variables that influence outcomes within the BFP method. Still, they were not considered criteria to obtain a considerable or minimum number of studies. These include culture media, concentrations and time procedures, optical density (OD) values and cut-offs, etc. The main purposes of the included papers were not to exclusively assess biofilm prevalence but also to link it with other factors or outcomes. Added to the fact that a single-arm analysis (lacking a comparison group) is not so valuable as two-arm, another problem is also noted, the use of in vitro methods as the only tool. In fact, they are the most used techniques to detect BFP capacity, but do not accurately represent in vivo conditions and are not always demonstrative of the biofilms found in infections [36].

#### 2.1.4. BFP Prevalence Related to Resistance: Two-Armed Meta-Analysis

A total of two papers were studied retrospectively, while for the other four, it was not mentioned. In only one paper, high BFP was considered prevalent (Supplementary Materials; S2. Study characteristics; Table S2).

**Figure 3:**
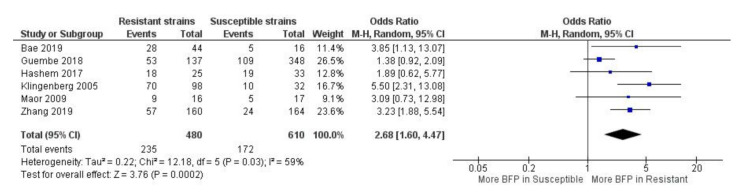
Forest plot of BFP (biofilm production) prevalence in resistant vs. susceptible strains—BSIs (Bloodstream Infections). “Tau^2^” represents the absolute value of the true variance (amount of heterogeneity). “Chi^2^” is the chi-squared test of the null hypothesis that there is no heterogeneity.”df” are degrees of freedom. “P” represents the *p*-value. The “I²” statistic describes the percentage of variation across studies that is due to heterogeneity rather than chance. “Z” is the z-statistic, which are significance tests for the weighted average effect size. “CI” stands for confidence interval. “M-H” is the Mantel-Haenszel analysis method.

Higher BFP prevalence in resistant strains was observed with high statistical significance (odds ratio-OR: 2.68; 95% CI: 1.60–4.47; *p* < 0.01) (Figure 3). A reasonable overlap is noticeable of CIs and OR estimates between all studies. This translated into a moderate statistical heterogeneity (I^2^ = 59%; *p* = 0.03), which is still remarkable since there is a sizeable heterogeneity in the study design (retrospective, retrospective cohort, prospective, resistance detection method, etc.) and clinical criteria (i.e., demographics, comorbidities, treatment interventions, outcomes, etc.) between studies. The observed results suggest that these factors do not seem to severely affect the influence of biofilm on antimicrobial resistance. Additionally, there was not a single study with an OR < 1, which would indicate higher BFP in susceptible strains. Thus, there is considerable evidence that biofilm may play an important role in the resistance to antibiotics by the identified strains. Yet, since five out of six studies reports were about methicillin-resistant *Staphylococcus* spp., this was mainly applicable to that group of bacteria.

Methicillin-resistant *S. aureus* (MRSA) is considered one of the most successful modern pathogen and represents a major threat to human health, with consistently high morbidity and mortality due to its ability to acquire resistance to most antibiotics commercially available [37]. It is transmitted in both healthcare and community settings, causing several other serious infections than BSI, such as endocarditis, skin/soft tissue infections, bone/joint infections, and ventilated assisted pneumonia. Methicillin-resistant *S. epidermidis* (MRSE) has emerged as a causative agent of infections often associated with implanted medical devices [38].

Resistance to methicillin antibiotic has been long and widely attributed to the presence of the *mecA* gene that encodes an enzyme called penicillin-binding protein 2a (PBP2a). This protein has a low affinity to *β*-lactam antibiotics, enabling cell wall synthesis in their presence [39,40,41]. The detection of methicillin-resistant strains can be done by phenotypic (such as oxacillin or cefoxitin disk diffusion) or genotypic methods (*mecA* gene detection) [40,42]. However, methicillin-resistant strain’s dependence on the *mecA* gene is so highly regarded by some researchers that its detection is a gold standard method. Phenotypic techniques may not detect strains with low expression or suppression of the *mecA* gene [43]. In this way, this procedure is usually used as a reference for the phenotypic detection of other genes [44,45,46,47,48].

Yet, the opposite situation is also alarming because methicillin resistance detected by phenotypic methods and undetected by genotypic ones might reflect other protection mechanisms (other than the PBP2a enzyme low affinity) that are often overlooked. In addition, common phenotypic methods do not detect specific biofilm resistance as they are performed with cells in a planktonic state. Fortunately, the view that methicillin resistance is exclusively related to the *mecA* gene has already been the subject of many studies, which reported the underestimations of the methicillin-resistant strains identified genotypically. In a study conducted in Sudan by Elhassan et al. [49], 123 MRSA isolates were detected phenotypically from different clinical specimens. From the identified MRSA, 12 (9.8%) were found to be *mecA* negative. Although considerable, this number was not impressive and the authors still recommended considering alternative methods to detect *β*-lactam resistance in order to find other mechanisms that can compete with the PBP2a mechanism and are related to the emergence of the MRSA phenomenon [42]. In Nigeria, Mustapha et al. [50] observed that all 80 MRSA strains identified and isolated from the nasal and perineal regions of dogs, were *mecA*-negative. In another study conducted in China by Wang and colaborators [51], from the total of bovine mastitis MRSA isolated strains 28 were *mecA*-negative and only six *mecA*-positive.

For the five considered papers in the present study (methicillin-resistant *Staphylococcus* spp.), Bae et al. [52], Hashem et al. [53] and Maor et al. [54] detected methicillin-resistant strains by phenotypic methods, Guembe et al. [55] did not mention, while Klingenberg et al. [56] identified them using *mecA* PCR detection procedure. Interestingly, this last study presented the highest OR (OR: 5.50; 95% CI: 2.31–13.08). In fact, BFP has been correlated to *mecA* positive methicillin-resistant *Streptococcus mutans*, MRSA and MRSE [57,58,59]. Côrtes et al. [57] have gone further in their investigation and BFP was also correlated to higher *mecA* transcription and PBP2a expression (for MRSA isolates). Apparently, the *mecA* gene can inactivate the accessory gene regulator (agr) quorum-sensing operon, increasing BFP. In addition, the repression of the agr in MRSA associated with mecA expression can promote alterations on the cell wall architecture, which is reflected by reduced production of cytotoxin and attenuation of the virulence [60]. Even though *mecA* presence or PBP2a expression induces higher BFP, biofilms and its protective mechanisms against antimicrobials should be more seriously considered as an independent impactful factor to methicillin resistance. For instance, a *mecA*-negative *S. aureus* strain isolated from bovine mastitis in Brazil, showed high resistance against the cefoxitin disk diffusion test and its resistance was linked with strong biofilms production [49]. Altogether, this evidence shows that methicillin resistance can be affected directly and/or indirectly by biofilms.

In the work of Zhang et al. [61] it was performed a retrospective study to infer the role of biofilm formation by *E. coli* extended-spectrum *β*-lactamases (ESBL) producers for cancer patients to develop BSI. They showed that ESBL *E. coli* (particularly *bla* _CTX-M-15_ type) are highly prevalent among cancer patients with BSI. In addition, the presence of biofilms contributes to the appearance of ESBL *E. coli* multi-drug resistance, increasing the risk of mortality in these patients. In fact, data obtained from the meta-analysis showed potentially worrying findings on the BFP impact, as it had a high OR and a comparatively narrow CI (OR: 3.23; 95% CI: 1.88–5.54). It is well known that the production of ESBLs is an important resistance mechanism that hampers the treatment of microbial infections [62]. It has already been observed that mortality rates associated with bacteremia caused by ESBL producing *E. coli* are significantly higher than those related with non-ESBL producing *E. coli* [63]. However, more data would be optimal to provide overall publications bias evaluation as well as to provide more confidence to assumptions.

#### 2.1.5. BFP Prevalence Related to Persistence: Two-Armed Meta-Analysis

For the BFP prevalence related to persistence, a total of three studies were retrospective and one was prospective, two were cohort and one multicenter. High BFP was considered prevalent in three studies, BF-positive (biofilm-positive) in one study and moderate/high metabolic activity in another one (Supplementary Materials; S2. Study characteristics; Table S3).

**Figure 4:**
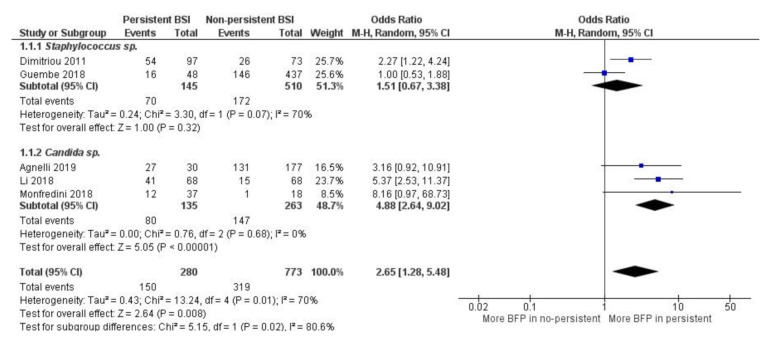
Forest plot of BFP (biofilm production) prevalence in persistent vs. non-persistent BSIs (Bloodstream Infections), and subgroup analysis by microorganism. “Tau^2^” represents the absolute value of the true variance (amount of heterogeneity). “Chi^2^” is the chi-squared test of the null hypothesis that there is no heterogeneity.”df” are degrees of freedom. “P” represents the *p*-value. The “I^2^” statistic describes the percentage of variation across studies that is due to heterogeneity rather than chance. “Z” is the z-statistic, which are significance tests for the weighted average effect size. “CI” stands for confidence interval. “M-H” is the Mantel-Haenszel analysis method.

Overall, high statistical significance indicates that BFP is related to persistent bacteremia/candidemia (OR: 2.65; 95% CI: 1.28–5.46; *p* < 0.01), and statistical heterogeneity was moderate (I^2^ = 70%; *p* = 0.01) (Figure 4). Once again, BFP impact on persistence can possibly be an independent factor from multiple study differences such as the resistance analysis (see Section 2.1.4). Data from all studies yielded OR values higher than one except for one paper.

Subgroup analysis indicates that there is significantly more BFP production in persistent candidemia (OR: 4.88; 95% CI: 2.64–9.02; *p* < 0.01) than in persistent bacteremia from *Staphylococcus* spp. (OR: 1.51; 95% CI: 0.67–3.38; *p* = 0.32). Within both subgroups, there was no heterogeneity. However, it was high between subgroups (I^2^ = 80.6%; *p* = 0.02), supporting the idea that *Candida* spp. biofilms can have a greater impact on infection persistence. Nevertheless, there is a substantial lack of studies, especially for subgroups analysis. Publication bias was not assessed.

Dimitriou et al. [64] demonstrated that only BFP was significantly linked to isolates from persistent bacteremia. On the other hand, Guembe et al. [55] (2018) only associated BFP with statistical significance to resistance (see Section 2.1.4), while association to persistence and mortality was not found (see Section 2.1.6).

In Agnelli et al. [65] and Li et al. [66], many factors were studied when comparing the persistent candidemia group vs. non-persistent candidemia (control group). In the first study, the authors did not associate BFP with inadequate therapeutic management. On the other hand, they found that BFP can be slightly correlated with infection persistence (*p* = 0.06) and higher “30-day mortality” rates, which means that the deaths occurred at 30th after hospital patient admission/infection detection (*p*= 0.18 and *p* = 0.28). In addition, in the second study, the authors also did not relate BFP to mortality, but strongly associated the retained central vascular catheters, the use of suboptimal doses of fluconazole and BFP with the development of persistent candidemia. Despite a large CI, the study of Monfredini et al. [67] was the one with the highest OR. They concluded that BFP should be considered an important biologic variable in explaining the failure to clear candidemia, despite regular antifungal treatment with triazoles.

#### 2.1.6. BFP Prevalence Related to Mortality: Two-Armed Meta-Analysis

**Figure 5:**
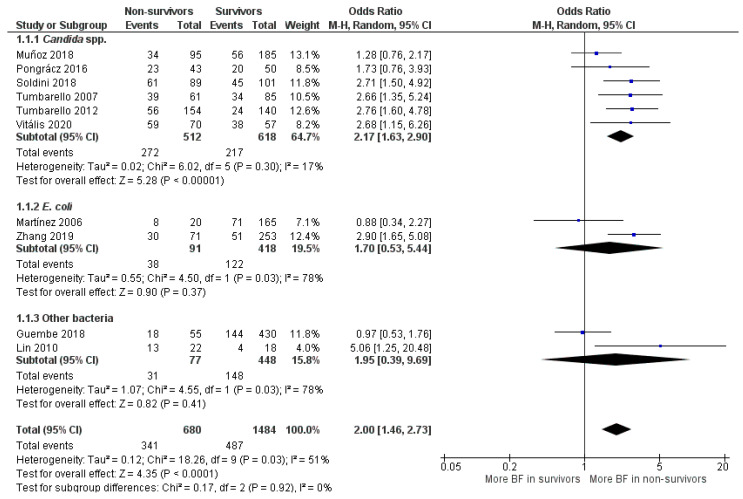
Forest plot of BFP (biofilm production) prevalence in BSIs (Bloodstream Infections) non-survivors vs. survivors and subgroup analysis by microorganism. “Tau^2^” represents the absolute value of the true variance (amount of heterogeneity). “Chi^2^” is the chi-squared test of the null hypothesis that there is no heterogeneity. “df” are degrees of freedom. “P” represents the *p*-value. The “I^2^” statistic describes the percentage of variation across studies that is due to heterogeneity rather than chance. “Z” is the z-statistic, which are significance tests for the weighted average effect size. “CI” stands for confidence interval. “M-H” is the Mantel-Haenszel analysis method.

For the BFP prevalence related to mortality of the 10 observational studies included, the majority were retrospective (*n* = 9), and only one was prospective. Two studies from the total were cohort. Seven studies described mortality as “30-day mortality” (after a defined event such as hospital admission or infection diagnosis), one paper as “14-day mortality” and two as “in-hospital mortality”. In five studies, BFP outcome prevalence was as BF-positive, three as high and moderate BFP and two as high BFP (Supplementary Materials; S2. Study characteristics; Table S4). Ten studies were included in the general meta-analysis (OR: 2.00; 95% CI: 1.46–2.73; *p* < 0.01), which was divided into three subgroups (Figure 5).

In “*E. coli*” (OR: 1.70; 95% CI: 0.53–5.44; *p* = 0.37) and “Other bacteria” subgroups (OR: 1.95; 95% CI: 0.39–9.69; *p* = 0.41), OR values leaned towards higher BFP in the non-survivors’ group, but statistical significance was not achieved. Statistical heterogeneity was high for both subgroups (I^2^ = 78%; *p* = 0.03) and they had one study each where OR values were close to 1, which made both estimates too uncertain (large CIs). Hence, these subgroups’ analyses are quite inconclusive due to insufficient papers and data. The most clarifying estimative was from the first subgroup (*Candida* spp.). High statistical significance indicates that BFP impacts candidemia mortality (OR: 2.05; 95% CI: 1.53–2.74), and there was no OR value below 1. Notably, only in Muñoz et al. [68] and Pongrácz et al. [69], the level of BFP did not correlate with statistical significance to candidemia mortality.

Candida BSI is widely recognized to cause significant morbidity and mortality. For example, a systematic review and meta-analysis carried by Koehler et al. [70] demonstrated that the estimated incidence of candidemia cases in Europe, for the period between January 2000 to February 2019, was 3.9 cases/100,000 population per year (literature search range; from the total of 43,799 cases in the mentioned period). Moreover, the estimated mortality rate was extremely high, with around 37% of “30-day mortality”. In another study performed by Rajendran et al. [71], a prospective analysis of patients with *Candida* BSI in the Scotland (total of 217 cases, between 2012–2013) revealed that BFP ability is significantly associated with *C. albicans* mortality (around 41% of “30-day mortality”). The authors further concluded that low biofilm formers and high biofilm formers respond differentially to antifungal therapy. Likewise, another systematic review conducted by Tsay et al. [72], estimated an incidence of 7.0 cases per 100,000 people, and “seven-day mortality” following candidemia detection was estimated to be approximately 15% (from the total of 1226 cases in the US in 2017). The application of combined antimicrobials and/or antifungals, and removal of catheters have routinely been practiced as a control measure, but they were not successful in eradicating BSIs [31]. It is believed that the burdens caused by *Candida* BSI can be mitigated by focusing more on the biofilm population or problem per se.

For the “*E. coli*” subgroup, two studies were considered. In the first study, Martínez et al. [73] conducted, in a prospective manner, their investigation for one year (Barcelona, Spain, 2003). Their goal was to assess the phylogenetic background, biofilm formation and time to detect the bacterial growth of the strains involved in a series of unselected/epidemiologically unrelated episodes of *E. coli* bacteremia. Therefore, all patients with bloodstream infections due to *E. coli* were included in their study. On the other hand, the second considered study was performed retrospectively at Tianjin (China) by Zhang and coworkers [61] for around four years and nine months (between January 2013 and September 2017). The goal was to investigate the impact of biofilm from *E. coli* BSI on the clinical outcome of hospitalized cancer patients, which are immunocompromised and more sensitive to virulence factors. Hence, beyond study type, mortality and BFP outcome descriptions differences (Supplementary Materials; S2. Study characteristics; Table S4), spatial-temporal and patient selection discrepancy can influence the results. Analyzing the study conclusions, while in the first work it was suggested that BFP does not foster the capability of *E. coli* to spread into the bloodstream. In the second one, it was claimed that BFP was an independent risk factor for mortality in hospitalized cancer patients with BSI.

Regarding subgroup “other bacteria”, in a similar way to what was observed with “*E. coli*” subgroup, the discrepancy of individual results is more expected when different bacteria are englobed. In the work of Guembe et al. [55] (2018), the association between BFP and poor clinical outcome in patients with *S. aureus* bacteremia was evaluated. They found that while a statistically significant correlation with methicillin resistance was found, the same was not verified with both persistence and mortality. In contrast, in the Lin et al. [74] studies, the high BFP by clinical isolates of *Chryseobacterium meningosepticum* was associated with cases of mortality in patients with bacteremia. They also concluded that the outcome of the patients was negatively affected by the choice of inadequate antimicrobial therapy and the use of long-term indwelling intravascular catheters. However, the small sample size was a clear limitation to the study.

Overall, the statistical heterogeneity score was moderate (I^2^ = 51%; *p* = 0.03), although the results obtained can be being influenced by the six *Candida* spp. studies, where the heterogeneity was much lower (I^2^ = 17%; *p* = 0.30). These low statistical values are remarkable since there is a lot of variance between the 6 included studies, with several significant description differences (study types, mortality and BFP outcome descriptions), as well as with other clinical heterogeneities. It may reveal that BFP impact on mortality has a very low dependence on those parameters (with treatment intervention included). This is particularly important to highlight because distinct contemporary treatment strategies or procedures do not seem to be tackling the biofilm issue in candidemia.

**Figure 6:**
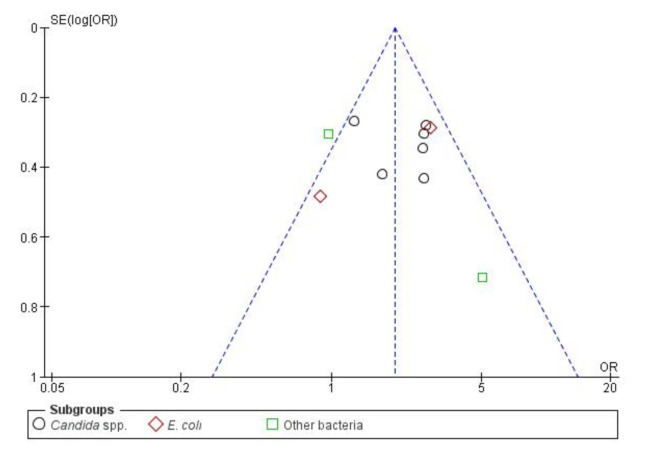
Funnel plot of standard error by OR (Odds Ratio)—BSIs (Bloodstream infections) resistance.

Publication bias was also evaluated, as 10 studies is the minimum recommended number to pool studies outcomes on a funnel plot (Figure 6). In the cases where there are fewer studies, the power of the tests is too low to distinguish chance from real asymmetry [75]. No major publication bias was detected as an acceptable symmetry is observed. Therefore, the integrity of the meta-analysis is assured.

### 2.2. Urinary Tract Infections

#### 2.2.1. Literature Search and Study Selection

**Figure 7:**
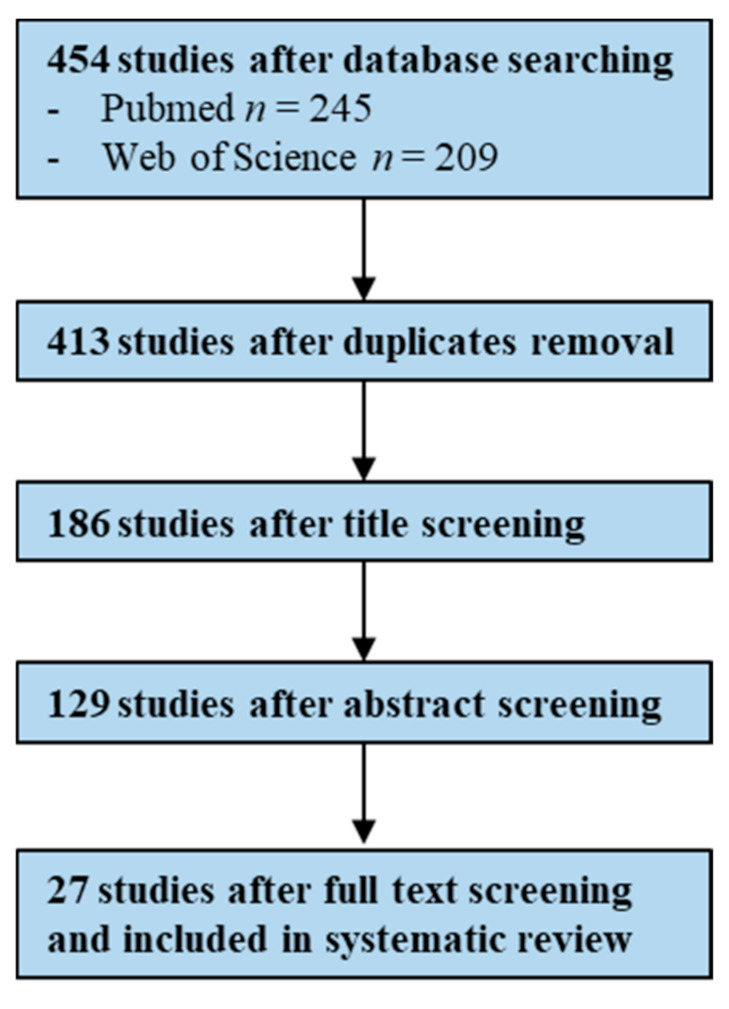
Flowchart illustrating the study screening process—UTIs (Urinary Tract Infections).

The systematic search on PubMed and Web of Science databases generated a total of 454 studies, of which 27 were identified as eligible after duplicates removal, title, abstract and full-text screening based on inclusion and exclusion criteria (Figure 7).

#### 2.2.2. Study Characteristics

Of the 27 eligible studies, BFP unrelated prevalence data were retrieved from 16 studies, 13 studies reported for BFP prevalence related to resistance and seven to CAUTI. Only one study had data eligible for all analysis, another single study for BFP unrelated prevalence and related to CAUTI, and a last single study for resistance and CAUTI. Five studies shared data for prevalence and resistance (Supplementary Materials; S2. Study characteristics; Tables S5, S6 and S7).

The pathogens reported in BFP unrelated prevalence analysis were: *E. coli* (*n* = 9), *Enterococcus* spp. (*n* = 3), *Klebsiella* spp. (*n* = 1), MRSA (*n* = 1), *Proteus* spp. (*n* = 1) and *A. baumanii* (*n* = 1). For prevalence related to resistance, the reported pathogens were: *E. coli* (*n* = 10), *Enterococcus* spp. (*n* = 1), *Klebsiella* spp. (*n* = 1) and MRSA (*n* = 1). For BFP prevalence related to CAUTI: *E. coli* (*n* = 4), *Enterococcus* spp. (*n* = 1), *E. faecalis* (*n* = 1) and Gram-negative *bacilli*/Gram-positive *cocci* (*n* = 1) (Supplementary Materials; S2. Study characteristics; Tables S5, S6 and S7).

#### 2.2.3. BFP Unrelated Prevalence: Single-Armed Meta-Analysis

**Figure 8:**
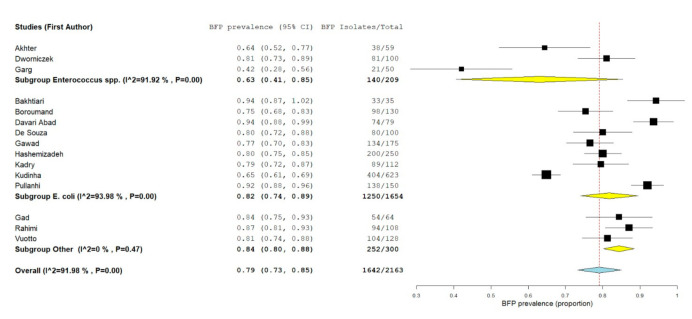
Forest plot of BFP (biofilm production) prevalence and subgroup analysis by microorganism—UTIs (Urinary Tract Infections). “P” represents the *p*-value. The “I^2^” statistic describes the percentage of variation across studies that is due to heterogeneity rather than chance, where “CI” stands for confidence interval.

Combined data from 16 studies were pooled in the forest plot presented in Figure 8. “*Enterococcus* spp.” subgroup had the lowest estimate proportion (proportion 0.63; 95% CI: 0.41–0.85; *p* < 0.01), followed by “*E. coli*” subgroup (proportion 0.82; 95% CI: 0.74–0.89; *p* < 0.01) and by “other” bacterial species subgroup (proportion 0.84; 95% CI: 0.80–0.88; *p* < 0.01). Overall BFP prevalence estimate was significantly higher (proportion 0.79; 95% CI: 0.73–0.85; *p* < 0.01) than the estimate observed in BSI analysis (see Section 2.1.3). However, statistical heterogeneity was very high (I^2^ = 92%; *p* < 0.01), just as it was in the BSI meta-analysis. This is once more, due to an absence of a fully standardized in vitro BFP method. In addition, the lack of a comparison group along with a simplistic (compared to in vivo) in vitro method, has led once again to inconclusive results. Statistical heterogeneity from the last subgroup (three studies from different bacterial species) was inexistent (I^2^ = 0%; *p* = 0.47), yet this seems to be a coincidence.

#### 2.2.4. BFP Prevalence Related to Resistance: Two-Armed Meta-Analysis

In BFP prevalence related to resistance analysis, a total of three papers were studied prospectively and one retrospectively (Supplementary Materials; S2. Study characteristics; Table S5). Four were cross-sectional studies and the other five did not specify. BF-positive were considered in all papers as prevalence, except for one where high/moderate BFP was considered instead (Supplementary Materials; S2. Study characteristics; Tables S2 and S6). Multi-drug resistant (MDR) microorganism was described as the resistance to three or more classes of antibiotics by all authors except for Karigoudar et al. [76] (two or more).

**Figure 9:**
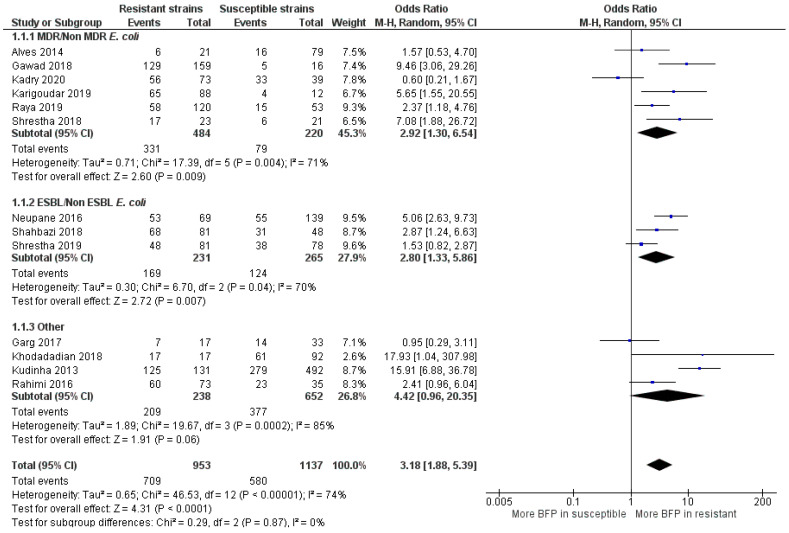
Forest plot of BFP prevalence in Resistant vs. Susceptible strains—UTIs (Urinary Tract Infections). “Tau^2^” represents the absolute value of the true variance (amount of heterogeneity). “Chi^2^” is the chi-squared test of the null hypothesis that there is no heterogeneity. “df” are degrees of freedom. “P” represents the *p*-value. The “I²” statistic describes the percentage of variation across studies that is due to heterogeneity rather than chance. “Z” is the z-statistic, which are significance tests for the weighted average effect size. “CI” stands for confidence interval. “M-H” is the Mantel-Haenszel analysis method.

Considering that UTI included more papers (*n* = 13) than in BSI meta-analysis (*n* = 6), high BFP prevalence in resistant strains was observed with slightly higher statistical significance (OR: 3.18; 95% CI: 1.88–5.39; *p* < 0.01) (Figure 9). However, contrary to BSI where overall statistical heterogeneity was low, UTI statistical heterogeneity was from moderate to high (I^2^ = 74%; *p* = 0.10). The reason for this divergence can be the higher number of studies, the higher difference in study designs and/or superior sensibility to clinical heterogeneities. The “other” subgroup heterogeneity was high (I^2^ = 85%; *p* < 0.01), while “MDR/Non-MDR *E. coli”* and “ESBL/non-ESBL *E. coli”* subgroup heterogeneities were both moderate (I^2^ = 71%; *p* < 0.01; I^2^ = 70%; *p* = 0.04), possibly reflecting the inclusion of the same group of *E. coli* resistance type.

MDR *E. coli* and ESBL *E. coli* had significant more BFP prevalence compared to non-MDR and non-ESBL, respectively (OR: 2.92; 95% CI: 1.30–6.54; *p* < 0.01 and OR: 2.80; 95% CI: 1.33–5.86; *p* < 0.01). A higher BFP prevalence in non-MDR *E. coli* (OR: 0.60; 95% CI: 0.21–1.67) was observed in one study. Kadry et al. [77] argued that biofilms provide secondary protection to uropathogenic *E. coli* and that the acquisition of resistance is not directly linked with biofilm formation. Without ruling out the potential impact of other factors than biofilms, the results from the meta-analysis clearly seem to contradict the authors and, indicate that they are usually not a secondary protection mechanism. Alves et al. [78] and Shrestha et al. [79] presented higher BFP in MDR and ESBL *E. coli*, respectively, but with no statistical significance (*p* = 0.39 and *p* = 0.21). However, they did not solely evaluate *E. coli*, but a group of different biofilm-producing bacteria isolated from patients with UTI, concluding that BFP strongly increases the resistance profile in a general manner. In summarizing important remarks from all studies, it is recommended biofilm screening, appropriated selection of antibiotics and a better understanding of the BFP ability, along with the development and application of new treatments and prevention strategies [76,78,80,81]

*E. coli* is by far the most causative UTI species, and with this meta-analysis, it was proven that biofilms can play a major role on the resistance rise of uropathogenic *E. coli* to currently used antimicrobials. Although the resistance augmentation phenomenon is already well documented and widespread, biofilms lack more recognition. For instance, many studies have already suggested the necessity for the careful monitoring of both antibiotic usage for UTI treatment and *E. coli* susceptibility [82,83,84,85]. However, in this work, it was demonstrated that the solution must go further and the implementation of new diagnostic, treatment and prevention strategies targeting biofilms in UTIs are essential.

The OR estimate for the “other” subgroup was extremely high (OR: 4.42; 95% CI: 0.96–20.35; *p* < 0.01). However, this value was leveraged by the outcome in the study of Kudinha et al. [86], as the OR value (OR: 15.91; 95% CI: 6.88–36.78) and sample size (*n* = 623) were enormous. In this work, BFP prevalence was compared in *E. coli* clonal group, sequence type 131 (ST131) vs. non ST131 *E. coli* isolates obtained from women of reproductive age (central west region of New South Wales-Australia) with cystitis or pyelonephritis. Even though no more studies reported ST131, BFP was strongly associated with its presence. ST131 is the predominant *E. coli* clonal group among extraintestinal pathogenic *E. coli* isolates worldwide, suggesting it contributes to resistance. They are frequently reported to produce ESBLs, and almost all are resistant to fluoroquinolones. Due to both the spectrum of infections that they cause and the large number of virulence-associated genes that they contain, ST131 *E. coli* isolates are considered to be truly pathogenic [87]. In this regard, Johnson et al. [88] reported that this clonal group is probably the cause of the most *E. coli* infections with associated resistance to antimicrobials in the US (2007). Garg et al. [89] did not recognize BFP as an independent factor for the observed resistance of *enterococci* to vancomycin isolated from UTIs. Khodadadian et al. [90] showed that the presence of *K. pneumoniae* producers of Metallo-*β*-lactamases in the samples of UTI (VIM-1 and IMP-1) are associated with BFP, despite a large CI shown in the meta-analysis. Finally, Rahimi et al. [91] highlighted the relevance of BFP and virulence factors expression by MRSA strains, in catheterized patients that developed UTI.

**Figure 10:**
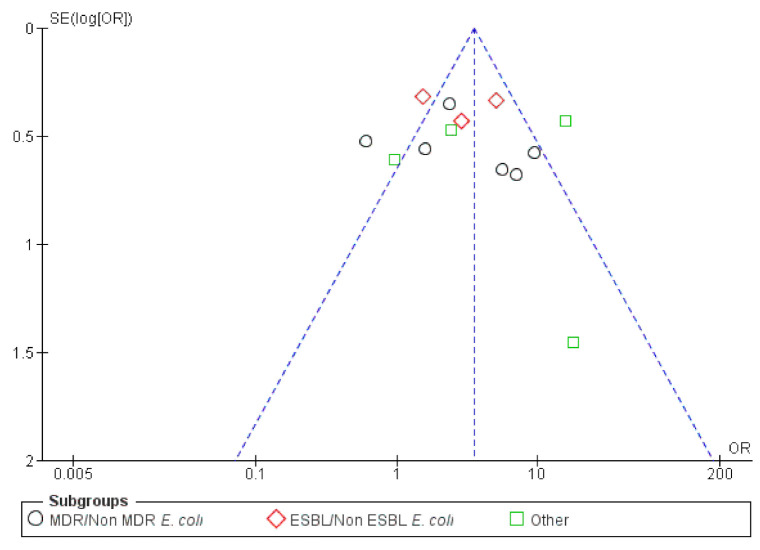
Funnel plot of standard error by OR (Odds ratio)—UTIs (Urinary Tract Infections) resistance.

Overall publication bias was assessed and the funnel plot is presented in Figure 10. A relatively balanced symmetry was observed and thereby, bias is undetected, the integrity of the meta-analysis was assured.

#### 2.2.5. BFP Prevalence Related to CAUTI: Two-Armed Meta-Analysis

**Figure 11:**
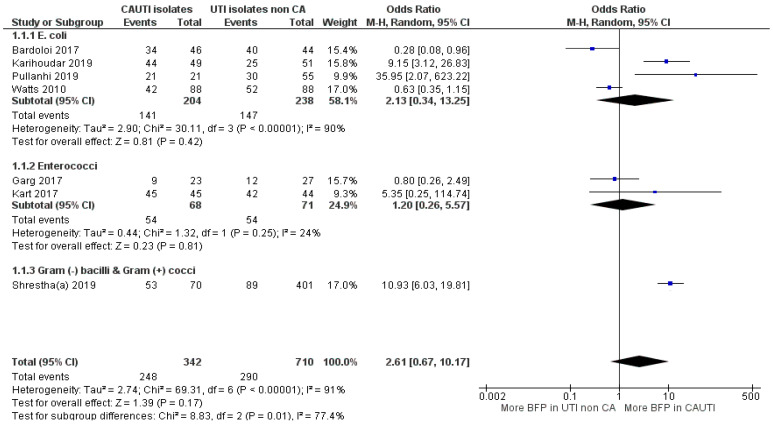
Forest plot of BFP (biofilm production) prevalence in CAUTI vs. non-CAUTI. “Tau^2^” represents the absolute value of the true variance (amount of heterogeneity). “Chi^2^” is the chi-squared test of the null hypothesis that there is no heterogeneity. “df” are degrees of freedom. “P” represents the *p*-value. The “I^2^” statistic describes the percentage of variation across studies that is due to heterogeneity rather than chance. “Z” is the z-statistic, which are significance tests for the weighted average effect size. “CI” stands for confidence interval. “M-H” is the Mantel-Haenszel analysis method.

In BFP prevalence related to CAUTI analysis, a total of three studies were conducted prospectively while the other three did not mention. BFP prevalence was shown to be BF-positive in all of them (Supplementary Materials; S2. Study characteristics; Table S7). For BFP prevalence in CAUTI vs. non-CAUTI, the meta-analysis was divided into two subgroups by bacterial species, which left a study reporting BFP prevalence on Gram-negative *bacilli* and Gram-positive *cocci* (Figure 11).

OR estimate from the “*Enterococcus* spp.” subgroup indicates a slight prevalence of BFP in CAUTI, but there was no statistical significance to confidently confirm (OR: 1.20; 95% CI: 0.26–5.57; *p* = 0.81). Only two studies were included, while one of them had an estimate under 1 OR, the other was too uncertain (large CI). The “*E. coli*” subgroup included four studies, but the obtained conclusions were not much different. Even though the OR estimate was significantly higher, the CI was too stretched towards 1 OR, due to two reports with securely more BFP in non-CAUTI. Overall, the “*E. coli*” subgroup statistical heterogeneities were high (I^2^ = 91%; *p* < 0.01 and I^2^ = 90%; *p* < 0.01; respectively), while the “*Enterococcus* spp.” subgroup (with only two studies) presented a low value (I^2^ = 24%; *p =* 0.25).

The study from Shrestha et al. [92] has a sizeable sample (*n* = 471) and a confident OR towards higher BFP in CAUTI. Consequently, it slightly weighted overall estimate into more prevalence in CAUTI, yet the confidence interval was not ideal (OR: 2.61; 95% CI: 0.67–10.17; *p* < 0.17). The authors not only observed significantly higher BFP in CAUTI than non-CAUTI, but the same was also verified with resistant strains. In fact, MDR (77% vs. 24%), MRSA (82% vs. 13%) and vancomycin-resistant *Enterococci* (33% vs. 3%) were much more prevalent in CAUTI than in non-CAUTI. However, it was not hypothesized that the higher BFP in CAUTI could have more connection to the strain’s resistance than to catheters. In contrast, Bardoloi and Yogeesha [93] presented the smallest association between BFP and CAUTI, while MDR *E. coli* rates between both groups were more balanced (89% in CAUTI vs. 80% in non-CAUTI). In their discussion, they claimed that attention should also be paid to the bacteria from community-acquired non-CAUTI that also have BFP ability. They also alleged that the in vitro BFP indicates the potential of the microorganism to produce biofilm but this cannot be directly extrapolated to in vivo.

Contrarily to other BAIs and non-CAUTIs, biofilms have been long accepted as the main and/or central attributable cause of CAUTIs [94]. From the global meta-analysis, it can be argued that the results were not expected to be so uncertain. However, despite CAUTI have been more directly impacted by biofilms, they can fairly impact non-CAUTI [95]. Moreover, the topology and characteristics of urinary catheters, the accumulation in the surface of fibrinogen following an immune response induced by catheterization, and the constant supply of nutrients from the urine stream, favours easy colonization of the microorganism and consequently biofilms establishment [24]. Thus, bacteria with less capacity to form in vitro biofilm, might be slightly abler to lead to biofilm formation on inserted catheters, reducing the gap towards strong in vitro biofilm producers.

In the general meta-analysis, the higher BFP prevalence, although non-statistically significant, is comprehensible. Therefore, it is unquestionably that the current focus on the search for new prevention and treatment strategies against biofilm formation on catheters, should not be distracted.

## 3. Materials and Methods

PRISMA for systematic review protocols (PRISMA-P) served as the main guide for conducting the systematic review and meta-analysis for both BSI and UTI [96].

### 3.1. Literature Search

A systematic review was carried out in both PubMed and Web of Sciences databases from January 2005 to May 2020, using a combination of Boolean operators (AND/OR/NOT), MeSH terms, publication types and other terms. Beyond several keyword combinations to identify BSIs or UTIs, the main keywords to identify biofilm prevalence and infection impact used were biofilm formation/presence/production; mortality; virulence; recurrence; persistence; morbidity; antibiotic resistance; risk factors; epidemiology; and clinical outcome/impact. Detailed search strategies are provided in the Supplementary Materials (1. Search terms and search strategies).

### 3.2. Study Selection

Papers were evaluated for eligibility (BSI—367 and UTI—454; BSI—312 and UTI—413, after duplicates removal), initially based on the title (BSI—154 and UTI—186), then on the abstract (BSI—94 and UTI—129) and finally on the full text (BSI—40 and UTI—27). Inclusion and exclusion criteria were individually predefined by H.P. and are presented in Table 1. H.P. is an MSc student that initiated to conduct this type of analysis during her thesis (with exclusive dedication along the semester).

Studies published before 2005 were not considered to ensure the focus on contemporary literature. With the intent to focus on standardized results, only studies that categorized data into positive/negative biofilm production (BFP) and performed crystal violet/safranin assay using microtiter/tissue culture plates with 24-h incubation, were included on the BFP prevalence single-arm analysis (no comparison group). These criteria were left out for BFP association with clinical outcomes or with resistant vs. susceptible strains (two-arm analyses) (see Section 3.4).

### 3.3. Data Extraction

Papers were retrieved from both databases and duplicates were removed using EndNote (X9.3.3, Clarivate Analytics). Data from eligible studies was extracted to a spreadsheet in Excel (Microsoft Office Excel 2016). The extracted data included first author, publication year, country, paper language, study type, bacteria, isolates origin, BFP detection method and sample size. Outcomes were divided into BFP prevalence (28 studies—BSI; 16 studies—UTI) and BFP in resistant vs. susceptible strains (BSI—6 studies; UTI—13 studies). Besides, they were divided into persistent vs. non-persistent (5 studies) and in survivors vs. non-survivor patients for BSI (10 studies); and CAUTI vs. non-CAUTI for UTI (6 studies). In some papers included in two-arm analyses, isolates were studied as high and/or moderate BFP vs. low BFP instead of BF-positive vs. BF-negative (Supplementary Materials; S2. Study characteristics). For two-arm analysis purposes, higher BFPs were considered as the BFP overall outcome.

Occasionally, data manipulation was necessary, and efforts were made to contact the authors when important data was missing. In addition, if *p*-values were not available within the studies, they were calculated (see Section 3.4). For studies reporting BFP in multiple microorganisms, only the group or species with the largest sample size was included whenever possible.

### 3.4. Data Analysis

Single-arm meta-analyses were conducted by H.P. using Open Meta [Analyst] software to determine overall BFP prevalence. The results were presented in proportion values (0 to 1). Two-arm meta-analyses were implemented using RevMan software (version 5.4, Cochrane) to determine BFP prevalence associated with resistance, persistence, mortality and CAUTI. The estimates were presented in odds ratio (OR). For both types of analysis, forest plots were generated using 95% confidence intervals (CI) to assess the significance of the results. When possible (*n* > 1), sub-group analysis was always undertaken by microorganism group or resistance type in the case of UTI. Statistical heterogeneities were calculated as I^2^ values, which were categorized as low (0–50%), moderate (50–75%), or high (>75%). A random-effects model was used to provide more confident data considering heterogeneity within and between reports. Studies were weighted in favor of those with thinner CIs. Publication bias was evaluated using the funnel plot when the number of studies was equal to or higher than 10. Missing *p*-values from data extraction were calculated as two-tailed values in GraphPad website, using a 2 × 2 contingency table and Fisher’s exact test [97].

## 4. Conclusions

BFP prevalence in both BSIs and UTIs was inconclusive as the single-arm meta-analyses were revealed to be inadequate. On the other hand, two-arm meta-analyses adequately suggested strong evidence that in general, BFP by microbial species impacts BSI resistance, persistence and mortality. Regarding microorganism sub-groups analysis, *Staphylococci* BFP had a significantly higher prevalence in resistant strains and *Candida* species BFP highly impacted mortality. The analyses of all other sub-groups of microorganisms showed potentially worrying findings, but there was insufficient data to properly assume BFP impact. There was also a lack of data to evaluate publication bias, except for the mortality impact since no major bias was detected. Regarding UTIs, the capability to BFP demonstrated to be substantially related to resistance, particularly for ESBL and MDR *E. coli* (the main uropathogen). ST131 clonal group showed very high BFP prevalence, despite data retrieved from only one study. Publication bias was not detected. On the other hand, BFP in CAUTI was not statistically significant. It can be hypothesised that isolates with less capability to form biofilms (detected by in vitro methods), could more easily be established in inserted catheters.

In terms of the limitations of the study, this review can be more prone to errors and individual bias since it was conducted by only one investigator. The number of papers included in each meta-analysis was not ideal for proper investigation, mainly due to a shortage of availability in the literature. As a matter of fact, during the selection process, some important aspects were noted. So, for further investigation, more observational studies are needed, especially concerning biofilm prevalence and its relation to clinical outcomes. More importantly than this are also the standardization of study designs and the applied methods. This would enable not only a collection of more eligible data but also lead to fewer heterogeneities. This strategy can also be applied to other specific biofilm implications.

For instance, there is a great number of studies trying to relate virulence genes and other biological assays to infer about biofilm infections. The main objective is to set researchers on the same side in order to seek faster and efficiently more concrete evidence. Conjugating systematic reviews of biofilm impact in a specific BAI with more socioeconomic variables can also be interesting (healthcare costs, geographical contrasts, etc.). Unfortunately, no more than one paper reporting BFP prevalence impact on UTI clinical outcomes, such as persistence and/or mortality was found. Besides, in the attempt to have a minimum or a reasonable number of studies, the criteria included in this study were not as many as the desirable (demographics, hospital setting, UTI type, comorbidities, treatment intervention, biofilm and resistance detection method, …), hindering the comparison of results.

Lastly, the majority of the observational studies included in both systematic reviews (BSIs and UTIs), referred that the microorganism identification was carried out by culture-based methods, which can lead to misleading data on the biofilm impact towards the involved microbial species. The replacement of culture techniques by molecular ones is urgent to better identify causative microbial species and understand their interactions. Indeed, biofilms are often composed of multiple microbial species, providing little-known dynamics that impact the infections differently from the single-species biofilms [98]. Although, the uses of culture techniques for the identification of the microorganisms associated with the infection be much more common, they are time-consuming, imprecise and sensitive to errors. These inaccuracies can be introduced by the specific growth conditions and culture media required by many fastidious microorganisms [99,100].

Advanced techniques started to be introduced in the routine clinical practices of the microbiological laboratories and include polymerase chain reaction (PCR), fluorescent in situ hybridization (FISH), microarrays, pyrosequencing and next-generation sequencing (NGS), mass spectrometry, T2 magnetic resonance (T2MR) and PCR electrospray ionization mass spectrometry (PCR/ESI-MS) [17,100].

Likewise, to accurately assess microorganism sensitivity in the sessile state, the implementation in clinical practices of susceptibility antimicrobial assays using biofilm cells is crucial. In this direction, microtiter plate-based methods have been developed and tested [101]. Calgary biofilm device, flow cell system and suspended substratum reactor are also promising for this kind of studies [101]. Parameters like minimal biofilm inhibitory concentration (MBIC), biofilm-prevention concentration (BPC), bactericidal biofilm concentration (BBC) and minimal biofilm-eradication concentration (MBEC) have been created as an alternative to the planktonic antimicrobial susceptibility parameters (minimum inhibitory concentration-MIC and minimum bactericidal concentration-MBC) [102]. Nonetheless, further efforts are needed in order to improve their accuracies, and thus, allow official agencies such as the “Clinical Laboratory Standard Institute (CLSI; Wayne- Delaware, Pennsylvania)” or the “European Committee on Antimicrobial Susceptibility Testing (EUCAST; Växjö, Sweden)” to standardize procedures, parameters and breakpoints [7,101,103].

The main mechanisms involved in biofilms recalcitrance toward antimicrobials and antifungals are multifactorial and complex [102,104,105]. Understanding biofilm processes is essential for the development of effective strategies to eradicate and/or control BAI [106,107,108]. Antibiotic or antifungal therapy alone often fails to eradicate biofilms. To date, there are no identified drugs directed to treat BAI [109]. In addition, the uncontrolled and inappropriate use of antibiotics contributes to the emergence of MDR bacteria [110]. It is expected that in the coming years, serious public health issues will arise if there is no drastic change in the use and development of new antibiotics [111]. Pharmaceutical companies are not prioritizing the discovery or invention of new antibiotics, as it is no longer seen as an economically wise investment [112]. Therefore, the need for new molecules and strategies for the treatment and prevention of BAI is urgent.

Favorably, in the recent past, an encouraging number of biofilm prevention and treatments strategies are emerging, which include: the coating of medical devices with polymers; the use of antibiotic adjuvants, quorum sensing inhibitors, adhesion block agents, biofilm dispersion agents and eradication/anti-persisters agents; and the implementation of phototherapy, antibiotic lock therapy and bacteriophage therapy [106,113,114,115,116]. Moreover, combining therapies and strategies can be extremely advantageous to encompass the different biofilm protective and colonization mechanisms. In this way, it is of relevance a strong collaboration, not only between researchers, medical and clinical staff but also among different disciplines (e.g., microbiology, surgery, internal medicine, pharmacology, biomaterials, nanotechnology and many more) [113,117]. Unfortunately, the majority of the mentioned strategies/agents are still in the very early stages of development, and a large investment of money and time are needed to convert them from laboratory tests to clinical trials and thus approval [108,115]. Likewise, the implementation of new diagnostic, treatment and prevention techniques will inevitably generate higher direct and indirect healthcare costs in the short term, by know-how requirements, the necessity of new routine introductions, devices, drugs and surgical procedures [118]. However, cost-effectiveness, in the long run, can be achieved if the burdens caused by BSI and UTIs are effectively tackled [118].

To conclude, tangible and high-level of evidence collected from systematic reviews can be a valuable incentive to push up the research focus on biofilm infections; encourage multidisciplinary collaboration; and incentive the investment and implementation by researchers, pharmaceuticals and official agencies of more accurate methods (e.g., molecular diagnostic techniques, biofilm antimicrobial susceptibility assays) as well as new strategies to treat and prevent BAI and its persistence. This study is an attempt to do this, trying to provide a clear picture of BSI and UTI burdens impacted by biofilms. To our knowledge, this is the first systematic review that analyses the biofilm impact on any BAI clinical outcome or antibiotic resistance.

## Figures and Tables

**Table 1:** Eligibility criteria for BSI (Bloodstream Infections) and UTI (Urinary Tract Infections).

Inclusion Criteria
- Observational study and original research
- Only Human BSI/bacteremia/fungemia/sepsis clinical isolates (BSI); Only Human UTI clinical isolates (UTI)
- Minimum of 15 clinical isolates (sample size)
- Isolates from blood cultures and/or catheter tips (BSI); Isolates from urine or catheters (UTI)
- Reports on biofilm in vitro production prevalence
- Reports on biofilm in vitro production prevalence related to clinical outcomes or to resistant vs. susceptible strains
- Healthcare settings (BSI); Healthcare settings (outpatients and inpatients) (UTI)
- In vitro biofilm production/detection only
- Crystal violet/safranin assay and on microtiter/tissue culture plates for biofilm production/detection *
- Biofilm formation in 24 h *
- Results in categorical data (Optical density (OD) cut-offs)
- OD cut-offs for negative/positive biofilm production *
- Studies published in English, French or Portuguese and from 1 January 2005
**Exclusion Criteria**
- Contaminant isolates
- Results in OD mean values

* For biofilm production prevalence only (one-arm study).

## Data Availability

Not applicable.

## Supplementary Materials

The following are available online at https://www.mdpi.com/article/10.3390/antibiotics10070825/s1; Table S1: Studies describing in vitro BFP prevalence in isolates from BSI patients; Table S2: Studies describing in vitro BFP prevalence in resistant and susceptible strains of isolates from BSI patients; Table S3; Studies describing in vitro BFP prevalence in isolates from persistent and non-persistent BSI. Table S5: Studies describing in vitro BFP prevalence in isolates from UTI patients. Table S6: Studies describing in vitro BFP prevalence in resistant and susceptible strains of isolates from UTI patients. Table S7: Studies describing in vitro BFP prevalence in isolates from CAUTI and UTI non-CAUTI.
