# Supplementary material for: Prevalence and Impact of Biofilms on Bloodstream and Urinary Tract Infections: A Systematic Review and Meta-Analysis
Source: Antibiotics (Basel). 2021 Jul 8;10(7):825. doi: 10.3390/antibiotics10070825 (PMC8300799; doi:10.3390/antibiotics10070825)
Supplement: Supplementary file 1 [file antibiotics-10-00825-s001.zip › antibiotics-1272501-SI.pdf]

antibiotics

MDPI

Article

Prevalence and Impact of Biofilms on Bloodstream and Urinary Tract Infections: A Systematic Review and Meta-Analysis

Henrique Pinto [1], Manuel Simões [1,2] and Anabela Borges [1,2,*]

[1] LEPABE - Laboratory for Process Engineering, Environment, Biotechnology and Energy, Faculty of Engineering, University of Porto, Rua Dr. Roberto Frias, s/n,, 4200-465 Porto, Portugal; up201708963@fe.up.pt (H.P.); mvs@fe.up.pt (M.S.)

[2] DEQ - Department of Chemical Engineering, Faculty of Engineering, University of Porto, Rua Dr. Roberto Frias, s/n, 4200-465 Porto, Portugal

* Correspondence: apborges@fe.up.pt; Tel.: +35-(122)-508-4968; Fax: +35-(122)-508-1449

1.Search Terms and Search Strategies

2.Study Characteristics

1.Search Terms and Search Strategies

1.1. Bloodstream Infections Systematic Review

1.1.1. PubMed Database Search

"((((biofilm[MeSH Terms]) OR (biofilm[Title])) AND (((((((((((((mortality) OR (hospital stay)) OR (length of stay)) OR (virulence)) OR (persistence)) OR (recurrence)) OR (biofilm formation)) OR (biofilm presence)) OR (biofilm incidence)) OR (biofilm prevalence)) OR (outcome)) OR (impact)) OR (risk factor)) OR (antibiotic resistance))) AND ((((((((((((((((bacteremia[MeSH Terms]) OR (candidemia[MeSH Terms])) OR (bacteraemia[MeSH Terms])) OR (candidaemia[MeSH Terms])) OR (bloodstream infections[MeSH Terms])) OR (catheter associated bacteremia[MeSH Terms])) OR (catheter related bacteremia[MeSH Terms])) OR (catheter associated bloodstream infections[MeSH Terms])) OR (cathether related bloodstream infections[MeSH Terms])) OR (catheter bloodstream infections[MeSH Terms])) OR (catheter bacteremia[MeSH Terms])) OR (bacteremia[Title])) OR (bacteraemia[Title])) OR (candidemia[Title])) OR (candidaemia[Title])) OR (bloodstream infection[Title])) OR (bloodstream infections[Title]))) NOT (((((((((((Review[Publication Type]) OR (Letter[Publication Type])) OR (Case reports[Publication Type])) OR (Meta-analysis[Publication Type])) OR (Editorial[Publication Type])) OR (Conference abstract[Publication Type])) OR (Correspondence[Publication Type])) OR (Comment[Publication Type])) OR (Systematic review[Publication Type] OR (Clinical trial[Publication Type] OR (randomized controlled trial[Publication Type]))))))) AND (2005:2020[pdat]) AND (english[Filter] OR french[Filter] OR portuguese[Filter])".

1.1.2. Web of Science Database Search

"TOPIC: (biofilm) *AND* **TITLE:** (mortality OR virulence OR recurrence OR persistence OR morbidity OR hospital stay OR length of stay OR antibiotic resistance) *AND* **TOPIC:** (bloodstream infections OR bacteremia OR bacteraemia OR candidemia OR candidaemia OR catheter related bloodstream infections OR catheter associated bloodstream infections) *NOT* **DOCUMENT TYPES:** (Review)

Refined by: DOCUMENT TYPES: (ARTICLE)".

1.2. Urinary Tract Infections Systematic Review

1.2.1. PubMed Database Search

"((((urinary catheter associated infections[MeSH Terms]) OR (urinary tract infections[MeSH Terms]) AND (english[Filter])) AND ((((biofilm[MeSH Terms]) OR (biofilm[Title])) OR (biofilms[MeSH Terms])) OR (biofilms[MeSH Terms]) AND (english[Filter]))) AND (((((((((mortality) OR (persistence)) OR (hospital)) OR (outcome)) OR (clinical)) OR (recurrence)) OR (risk factor)) OR (resistance) OR (impact) OR (epidemiology) OR (production) AND (english[Filter])))) NOT ((((((((((((((Review[Publication Type]) OR (Letter[Publication Type])) OR (Case reports[Publication Type])) OR (Meta-analysis[Publication Type])) OR (Editorial[Publication Type])) OR (Conference abstract[Publication Type])) OR (Correspondence[Publication Type])) OR (Comment[Publication Type])) OR (Systematic review[Publication Type] OR (Clinical trial[Publication Type] OR (randomized controlled trial[Publication Type])))))))) AND (english[Filter])) AND ((english[Filter]) AND (2005:2020[pdat])) AND (english[Filter]) AND (english[Filter]) Filters: English, French, Portuguese".

1.2.2. Web of Science Database Search

"TOPIC: (biofilm) *AND* **TITLE:** (mortality OR recurrence* OR persist* OR hospital* OR outcome OR clinical OR resist* OR impact* OR epidemiology OR risk factors OR production) *AND* **TOPIC:** (urinary tract infections OR urinary catheter associated infections) *NOT* **DOCUMENT TYPES:** (Review)".

2. Study Characteristics

Table S1. Studies Describing in vitro BFP prevalence in isolates from BSI patients.

**Study (Reference)**	**Country**	**Study type**	**Microorganism**	**BFP prevalence** n/N (%)
[1]	Turkey	NS	*Candida* spp.	8/50 (16%)
[2]	France	Prospective cohort	*E. coli*	67/53 (43.8%)
[3]	Spain	NS	*E. faecalis*	18/22 (81.8%)
[4]	Greece	NS	CoNS	58/100 (58.0%)
[5]	Spain	Retrospective	*S. aureus*	162/323 (50.1%)
[6]	Spain	Retrospective	*Candida* spp.	45/54 (83.3%)
[7]	Egypt	NS	*Staphylococcus* spp.	37/58 (63.8%)
[8]	Brazil	NS	*S. aureus*	25/40 (62.5%)
[9]	Norway	NS	CoN *S. epidermidis*	79/130 (60.8%)
[10]	Israel	NS	*S. aureus*	14/33 (42.4%)
[11]	Spain	Prospective	*E. coli*	79/185 (42.7%)
[12]	Turkey	NS	*Candida* spp.	15/52 (28.8%)
[13]	South Africa	Prospective	MSSA	13/21 (61.9%)
[14]	France	Retrospective	*S. epidermidis*	59/98 (60.2%)
[15]	Greece	Retrospective	CoN *S. epidermidis*	14/19 (73.7%)

[16]	Brazil	NS	CoNS	59/59 (100%)
[17]	Brazil	NS	MR *S.haemolyticus*	23/24 (95.8%)
[18]	Brazil	NS	*S. haemolyticus*	39/48 (81,3%)
[19]	Hungary	Retrospective	*Candida* spp.	43/93 (46.2%)
[20]	Austria	NS	Viridians *Streptococci*	6/22 (27.3%)
[21]	Austria	Prospective	*S. epidermidis*	53/60 (88.3%)
[22]	Italy	NS	*C. parapsilosis*	31/31 (100%)
[23]	Japan	NS	MR *Corynebacterium* spp.	14/17 (82.4%)
[24]	Brazil	NS	CoNS	72/176 (40.9%)
[25]	Slovenia	NS	*E. coli*	55/105 (52.3%)
[26]	Brazil	NS	*S. epidermidis*	8/31 (25.8%)
[27]	Thailand	NS	*Candida* spp.	58/84 (69.0%)
[28]	China	Retrospective	*E. coli*	81/324 (25.0%)

Notes: NS: Not specified; E. coli: Escherichia coli; E. faecalis: Enterococcus faecalis; CoNS: Coagulase-negative Staphylococci; CoN: Coagulase-negative; S. aureus: Staphylococcus aureus; S. epidermidis: Staphylococcus epidermidis; MSSA: Methicillin-susceptible Staphylococcus aureus; MR: Methicillin-resistant; S. haemolyticus: Staphylococcus haemolyticus; C. parapsilosis: Candida parapsilosis.

Table S2. Studies describing in vitro BFP prevalence in resistant and susceptible strains of isolates from BSI patients.

**Study (Reference)**	**Country**	**Study type**	**Microorganism**	**BFP prevalence** n/N (%)	***p*-value**
[29]	South Korea	NS	MRSA	28/44 (63.6%)	0.040 *
			MSSA	5/16 (31.3%)	
[5]	Spain	Retrospective	MRSA	53/137 (38.7%) **	0.135 *
			MSSA	109/348 (31.3%) **	
[7]	Egypt	NS	MR *Staphylococcus* spp.	18/25 (72.0%)	0.285 *
			MS *Staphylococcus* spp.	19/33 (57.6%)	
[9]	Norway	NS	CoN MRSE	70/98 (71.4%)	<0.001
			CoN MSSE	9/32 (28.1%)	
[10]	Israel	NS	MRSA	9/16 (55.5%)	0.166 *
			MSSA	5/17 (29%)	
[28]	China	Retrospective	ESBL *E. coli*	57/160 (35.6%)	<0.001

			Non ESBL *E. coli*	24/164 (14.6%)	

Notes: *Missing *p*-value from data extraction were calculated; **only High BFP; NS: Not specified; MRSA: Methicillin-resistant *Staphylococcus aureus;* MSSA: Methicillin-susceptible *Staphylococcus aureus;* CoN: Coagulase-negative; MRSE: Methicillin-resistant *Staphylococcus epidermidis;* MSSE: Methicillin-susceptible *Staphylococcus epidermidis;* ESBL: Extended-spectrum beta-lactamase; *E. coli*: *Escherichia coli*.

Table S3. Studies describing in vitro BFP prevalence in isolates from persistent and non-persistent BSI.

Study (Reference)	Country	Study type	Microorganism	BFP outcome description	**BFP prevalence** n/N (%)		*p*-value
					Persistent BSI	Non-persistent BSI	
[30]	Spain	Retrospective cohort	*Candida* spp.	Moderate/high metabolic activity	27/30 (90.0%)	131/177 (74.0%)	0.064
[31]	Greece	Prospective	CoN *Staphylococci*	BF positive	54/97 (55.7%)	26/73 (35.6%)	0.013
[5]	Spain	Retrospective	*S. aureus*	Only High BFP	16/48 (33.3%)	146/437 (33.4%)	1.000 *
[32]	Taiwan	Retrospective	*Candida* spp.	Only High BFP	41/68 (60.3%)	15/68 (22.1%)	<0.010
[33]	Brazil	Multicenter cohort	*Candida* spp.	Only High BFP	12/37 (32.4%)	1/18 (5.5%)	0.041 *

Notes: *Missing *p*-value from data extraction were calculated; CoN: Coagulase-negative; *S. aureus: Staphylococcus aureus* .

Table S4. Studies describing in vitro BFP prevalence in isolates of BSI survivors and non-survivors.

Study (Reference)	Country	Study type	Microorganism	Mortality description	BFP outcome description	**BFP prevalence** n/N (%)		*p*-value
						BSI non-survivors	BSI survivors	
[5]	Spain	Retrospective	*S. aureus*	30-day mortality	Only High BFP	18/55 (32.7%)	144/430 (33.5%)	1.000 *
[34]	Taiwan	Retrospective	*C. meningosepticum*	14-day mortality	BF-positive	13/22 (59.1%)	4/18 (22.2%)	0.019
[11]	Spain	Prospective	*E. coli*	In-hospital	BF-positive	8/20 (40.0%)	71/165 (43.0%)	0.800
[35]	Spain	Retrospective cohort	*Candida* spp.	30-day mortality	Only high BFP	34/95 (35.8%)	56/185 (30.3%)	0.418
[19]	Hungary	Retrospective	*Candida* spp.	30-day mortality	BF-positive	23/43 (53.5%)	20/50 (40%)	0.216 *
[36]	Italy	Retrospective	*C. parapsilosis*	30-day mortality	High and moderate BFP	61/89 (68.5%)	45/101 (44.6%)	0.010

[37]	Italy	Retrospective	*Candida* spp.	In-hospital	BF-positive	39/61 (63.9%)	34/85 (40.0%)	0,010
[38]	Italy	Retrospective cohort	*Candida* spp.	30-day mortality	BF-positive	56/154 (36.3%)	24/140 (17.1%)	<0.001
[39]	Hungary	Retrospective	*Candida* spp.	30-day mortality	High and moderate BFP	59/70 (84.3%)	38/57 (66.7%)	0.023
[28]	China	Retrospective	*E. coli*	30-day mortality	High and moderate BFP	30/71 (42.2%)	51/253 (20.2%)	0.002

Notes: *Missing p-value from data extraction were calculated; C. meningosepticum: Corynebacterium meningosepticum; E. coli: Escherichia coli; C. parapsilosis: Candida parapsilosis.

Table S5. Studies describing in vitro BFP prevalence in isolates from UTI patients.

**Study (Reference)**	**Country**	**Study type**	**Microorganism**	**BFP prevalence** n/N (%)
[40]	Bangladesh	NS	*Enterococcus* spp.	76/118 (64.4%)
[41]	Iran	NS	*E. coli*	33/35 (94.3%)
[42]	Iran	Cross Sectional	*E. coli*	98/130 (75.4%)
[43]	Iran	Cross Sectional	*E. coli*	74/79 (93.7%)
[44]	Brazil	NS	*E. coli*	80/100 (80.0%)
[45]	Poland	NS	*Enterococci*	81/100 (81%)
[46]	Egypt	NS	*Klebsiella* spp.	54/64 (84.4%)
[47]	India	Prospective	*Enterococcus* spp.	21/50 (42.0%)
[48]	Egypt	NS	*E. coli*	134/175 (76.6%)
[49]	Iran	NS	*E. coli*	200/250 (80.0%)
[50]	Egypt	NS	*E. coli*	89/112 (79.5%)
[51]	Australia	Prospective	*E. coli*	404/623 (64.8%)
[52]	India	NS	*E. coli*	138/150 (92.0%)
[53]	Iran	NS	MR *Staphylococcus* spp.	94/108 (59.3%)
[54]	Iran	Retrospective	*Proteus* spp.	82/88 (93.2%)
[55]	Europe *	NS	*Acinetobacter baumannii*	104/128 (81.3%)

Notes: *5 countries; NS: Not specified; *E. coli*: *Escherichia coli*; MR: Methicillin-resistant.

Table S6. Studies describing in vitro BFP prevalence in resistant and susceptible strains of isolates from UTI patients.

Study (Reference)	Country	Study type	Microorganism	BFP prevalence n/N (%)	*p*-value
[56]	Portugal	Retrospective	MDR *E. coli*	6/21 (28.6%)	0.393 *
			Non MDR *E. coli*	16/79 (20.2%)	
[47]	India	Prospective	VRE	7/17 (41.2%)	1.000 *
			VSE	14/33 (42.4%)	
[48]	Egypt	NS	MDR *E. coli*	129/159 (81.1%)	<0.001
			Non MDR *E. coli*	5/16 (31.3%)	
[50]	Egypt	NS	MDR *E. coli*	56/73 (76.7%)	0.462 *
			Non MDR *E. coli*	33/39 (84.6%)	
[57]	India	NS	MDR *E. coli*	65/88 (73.9%)	0.001
			Non MDR *E. coli*	4/12 (33.3%)	
[58]	Iran	Cross Sectional	MBL *K. pneumoniae*	17/17 (100%) **	0.003
			N-MBL *K. pneumoniae*	61/92 (66.3%) **	
[51]	Australia	Prospective	st131 *E. coli*	125/131 (95.4%)	<0.001
			Non st131 *E. coli*	279/492 (56.7%)	
[59]	Nepal	Cross Sectional	ESBL *E. coli*	53/69 (76.8%)	0.050
			Non ESBL *E. coli*	55/139 (39.6%)	
[53]	Iran	NS	MDR MRSA	60/73 (82.2%)	0.086 *
			Non MDR MRSA	23/35 (65.7%)	
[60]	Nepal	Cross Sectional	MDR *E. coli*	58/120 (48.3%)	0.015
			Non MDR *E. coli*	15/53 (28.3%)	
[61]	Iran	NS	ESBL *E. coli*	68/81 (83.9%)	0.010 *
			Non ESBL *E. coli*	31/48 (64.6%)	
[62]	Nepal	Prospective	MDR *E. coli*	17/23 (73.9%)	0.006 *
			Non MDR *E. coli*	6/21 (28.6%)	
[63]	Nepal	Cross Sectional	ESBL *E. coli*	48/81 (59.3%)	0.205 *
			Non ESBL *E. coli*	38/78 (48.7%)	

Notes: *Missing *p*-value from data extraction were calculated; **only High/moderate BFP; NS: Not specified; MRSA: Methicillin-resistant *Staphylococcus aureus;* ESBL: Extended-spectrum beta-lactamase; *E. coli*: *Escherichia coli*; MDR: Multi-drug resistant; VRE: *Vancomycin-Resistant Enterococci; VSE: Vancomycin-Susceptible Enterococci; MBL: Metallo-Beta-Lactamase; K. pneumoniae: Klebsiella pneumoniae.*

Table S7. Studies describing in vitro BFP prevalence in isolates from CAUTI and UTI non-CAUTI.

**Study (Reference)**	**Country**	**Study type**	**Microorganism**	**BFP prevalence** n/N (%)		***p*-value**
				CAUTI	UTI non-CA	
[64]	India	Prospective	*E. coli*	34/46 (73.9%)	40/44 (90.9%)	0.052 *
[47]	India	Prospective	*Enterococcus* spp.	9/23 (39.1%)	12/27 (44.4%)	0.779 *
[57]	India	NS	*E. coli*	44/49 (89.7%)	25/51 (49.0%)	<0.001 *
[52]	India	NS	*E. coli*	21/21 (100%)	30/55 (54.5%)	<0.001*
[65]	Nepal	Prospective	Gram (-) bacilli & Gram (+) cocci	53/70 (75.7%)	89/401 (22.2%)	<0.001 *
[66]	Aus-tralia	NS	*E. coli*	42/88 (47.7%)	52/88 (59.1%)	0.174

Notes: Only BF-positive; *Missing *p*-value from data extraction were calculated; *E. coli*: *Escherichia coli;* Gram (-): Gram-negative; Gram (+): Gram-positive.

References

1. Atalay MA, Koc AN, Demir G, Sav H. Investigation of possible virulence factors in *Candida* strains isolated from blood cultures. *Niger J Clin Pract* 2015;**18**(1):52-5. doi: 10.4103/1119-3077.146979.
2. Buffet-Bataillon S, Branger B, Cormier M, Bonnaure-Mallet M, Jolivet-Gougeon A. Effect of higher minimum inhibitory concentrations of quaternary ammonium compounds in clinical *E. coli* isolates on antibiotic susceptibilities and clinical outcomes. *Journal of Hospital Infection* 2011;**79**(2):141-6. doi: 10.1016/j.jhin.2011.06.008.
3. Cafini F, Gómez-Aguado F, Corcuera MT, Ramos C, Bas P, Collado L, et al. Genotypic and phenotypic diversity in *Enterococcus faecalis*: is agar invasion a pathogenicity score? *Rev Esp Quimioter* 2015;**28**(2):101-8.
4. Giormezis N, Kolonitsiou F, Foka A, Drougka E, Liakopoulos A, Makri A, et al. Coagulase-negative *staphylococcal* bloodstream and prosthetic-device-associated infections: the role of biofilm formation and distribution of adhesin and toxin genes. *J Med Microbiol* 2014;**63**(Pt 11):1500-8. doi: 10.1099/jmm.0.075259-0.
5. Guembe M, Alonso B, Lucio J, Pérez-Granda MJ, Cruces R, Sánchez-Carrillo C, et al. Biofilm production is not associated with poor clinical outcome in 485 patients with *Staphylococcus aureus* bacteraemia. *Clin Microbiol Infect* 2018;**24**(6):659.e1-.e3. doi: 10.1016/j.cmi.2017.10.018.
6. Guembe M, Guinea J, Marcos-Zambrano L, Fernández-Cruz A, Peláez T, Muñoz P, et al. Is biofilm production a predictor of catheter-related candidemia? *Med Mycol* 2014;**52**(4):407-10. doi: 10.1093/mmy/myt031.
7. Hashem AA, Abd El Fadeal NM, Shehata AS. In vitro activities of vancomycin and linezolid against biofilm-producing methicillin-resistant *staphylococci* species isolated from catheter-related bloodstream infections from an Egyptian tertiary hospital. *J Med Microbiol* 2017;**66**(6):744-52. doi: 10.1099/jmm.0.000490.
8. Iorio NL, Lopes AP, Schuenck RP, Barcellos AG, Olendzki AN, Lopez GL, et al. A combination of methods to evaluate biofilm production may help to determine the clinical relevance of *Staphylococcus* in blood cultures. *Microbiol Immunol* 2011;**55**(1):28-33. doi: 10.1111/j.1348-0421.2010.00288.x.
9. Klingenberg C, Aarag E, Rønnestad A, Sollid JE, Abrahamsen TG, Kjeldsen G, et al. Coagulase-negative *staphylococcal* sepsis in neonates. Association between antibiotic resistance, biofilm formation and the host inflammatory response. *Pediatr Infect Dis J* 2005;**24**(9):817-22. doi: 10.1097/01.inf.0000176735.20008.cd.
10. Maor Y, Lago L, Zlotkin A, Nitzan Y, Belausov N, Ben-David D, et al. Molecular features of heterogeneous vancomycin-intermediate *Staphylococcus aureus* strains isolated from bacteremic patients. *BMC Microbiol* 2009;**9**:189. doi: 10.1186/1471-2180-9-189.
11. Martínez JA, Soto S, Fabrega A, Almela M, Mensa J, Soriano A, et al. Relationship of phylogenetic background, biofilm production, and time to detection of growth in blood culture vials with clinical variables and prognosis associated with *Escherichia coli* bacteremia. *J Clin Microbiol* 2006;**44**(4):1468-74. doi: 10.1128/jcm.44.4.1468-1474.2006.
12. Mutlu Sariguzel F, Berk E, Koc AN, Sav H, Demir G. Investigation of the relationship between virulence factors and genotype of *Candida* spp. isolated from blood cultures. *J Infect Dev Ctries* 2015;**9**(8):857-64. doi: 10.3855/jidc.5359.
13. Naicker PR, Karayem K, Hoek KG, Harvey J, Wasserman E. Biofilm formation in invasive *Staphylococcus aureus* isolates is associated with the clonal lineage. *Microb Pathog* 2016;**90**:41-9. doi: 10.1016/j.micpath.2015.10.023.
14. Ninin E, Caroff N, Espaze E, Maraillac J, Lepelletier D, Milpied N, et al. Assessment of ica operon carriage and biofilm production in *Staphylococcus epidermidis* isolates causing bacteraemia in bone marrow transplant recipients. *Clin Microbiol Infect* 2006;**12**(5):446-52. doi: 10.1111/j.1469-0691.2006.01382.x.

15. Papadimitriou-Olivgeri I, Giormezis N, Papadimitriou-Olivgeris M, Zotou A, Kolonitsiou F, Koutsileou K, et al. Number of positive blood cultures, biofilm formation, and adhesin genes in differentiating true coagulase-negative *staphylococci* bacteremia from contamination. *Eur J Clin Microbiol Infect Dis* 2016;**35**(1):57-66. doi: 10.1007/s10096-015-2506-7.
16. Pedroso S, Sandes SHC, Luiz KCM, Dias RS, Filho RAT, Serufo JC, et al. Biofilm and toxin profile: a phenotypic and genotypic characterization of coagulase-negative staphylococci isolated from human bloodstream infections. *Microb Pathog* 2016;**100**:312-8. doi: 10.1016/j.micpath.2016.10.005.
17. Pereira PM, Binatti VB, Sued BP, Ramos JN, Peixoto RS, Simões C, et al. *Staphylococcus haemolyticus* disseminated among neonates with bacteremia in a neonatal intensive care unit in Rio de Janeiro, Brazil. *Diagn Microbiol Infect Dis* 2014;**78**(1):85-92. doi: 10.1016/j.diagmicrobio.2013.06.026.
18. Pereira-Ribeiro PM, Sued-Karam BR, Faria YV, Nogueira BA, Colodette SS, Fracalanzza SE, et al. Influence of antibiotics on biofilm formation by different clones of nosocomial *Staphylococcus haemolyticus*. *Future Microbiol* 2019;**14**:789-99. doi: 10.2217/fmb-2018-0230.
19. Pongrácz J, Benedek K, Juhász E, Iván M, Kristóf K. In vitro biofilm production of *Candida* bloodstream isolates: any association with clinical characteristics? *J Med Microbiol* 2016;**65**(4):272-7. doi: 10.1099/jmm.0.000207.
20. Presterl E, Grisold AJ, Reichmann S, Hirschl AM, Georgopoulos A, Graninger W. *Viridans streptococci* in endocarditis and neutropenic sepsis: biofilm formation and effects of antibiotics. *J Antimicrob Chemother* 2005;**55**(1):45-50. doi: 10.1093/jac/dkh479.
21. Presterl E, Lassnigg A, Parschalk B, Yassin F, Adametz H, Graninger W. Clinical behavior of implant infections due to *Staphylococcus epidermidis*. *Int J Artif Organs* 2005;**28**(11):1110-8. doi: 10.1177/039139880502801108.
22. Pulcrano G, Panellis D, De Domenico G, Rossano F, Catania MR. Ambroxol influences voriconazole resistance of *Candida parapsilosis* biofilm. *FEMS Yeast Res* 2012;**12**(4):430-8. doi: 10.1111/j.1567-1364.2012.00792.x.
23. Qin L, Sakai Y, Bao R, Xie H, Masunaga K, Miura M, et al. Characteristics of multidrug-resistant *Corynebacterium* spp. isolated from blood cultures of hospitalized patients in Japan. *Jpn J Infect Dis* 2017;**70**(2):152-7. doi: 10.7883/yoken.JJID.2015.530.
24. Rampelotto RF, Lorenzoni VV, Silva DDC, Coelho SS, Wust V, Garzon LR, et al. Assessment of different methods for the detection of biofilm production in coagulase-negative *staphylococci* isolated from blood cultures of newborns. *Rev Soc Bras Med Trop* 2018;**51**(6):761-7. doi: 10.1590/0037-8682-0171-2018.
25. Rijavec M, Müller-Premru M, Zakotnik B, Žgur-Bertok D. Virulence factors and biofilm production among *Escherichia coli* strains causing bacteraemia of urinary tract origin. *J Med Microbiol* 2008;**57**(Pt 11):1329-34. doi: 10.1099/jmm.0.2008/002543-0.
26. Salgueiro VC, Iorio NLP, Ferreira MC, Chamon RC, Dos Santos KRN. Methicillin resistance and virulence genes in invasive and nasal *Staphylococcus epidermidis* isolates from neonates. *BMC microbiology* 2017;**17**(1):15-. doi: 10.1186/s12866-017-0930-9.
27. Sriphannam C, Nuanmuang N, Saengsawang K, Amornthipayawong D, Kummasook A. Anti-fungal susceptibility and virulence factors of *Candida* spp. isolated from blood cultures. *Journal de Mycologie Médicale* 2019;**29**(4):325-30. doi: 10.1016/j.mycmed.2019.08.001.
28. Zhang Q, Gao HY, Li D, Li Z, Qi SS, Zheng S, et al. Clinical outcome of *Escherichia coli* bloodstream infection in cancer patients with/without biofilm formation: a single-center retrospective study. *Infect Drug Resist* 2019;**12**:359-71. doi: 10.2147/idr.S192072.
29. Bae E, Kim CK, Jang JH, Sung H, Choi Y, Kim MN. Impact of community-onset methicillin-resistant *Staphylococcus aureus* on *Staphylococcus aureus* bacteremia in a central Korea veterans health service hospital. *Ann Lab Med* 2019;**39**(2):158-66. doi: 10.3343/alm.2019.39.2.158.
30. Agnelli C, Valerio M, Bouza E, Vena A, Guinea J, Del Carmen Martínez-Jiménez M, et al. Persistent candidemia in adults: underlying causes and clinical significance in the antifungal stewardship era. *Eur J Clin Microbiol Infect Dis* 2019;**38**(3):607-14. doi: 10.1007/s10096-019-03477-3.
31. Dimitriou G, Fouzas S, Giormezis N, Giannakopoulos I, Tzifas S, Foka A, et al. Clinical and microbiological profile of persistent coagulase-negative *staphylococcal* bacteraemia in neonates. *Clin Microbiol Infect* 2011;**17**(11):1684-90. doi: 10.1111/j.1469-0691.2011.03489.x.
32. Li WS, Chen YC, Kuo SF, Chen FJ, Lee CH. The impact of biofilm formation on the persistence of candidemia. *Front Microbiol* 2018;**9**:1196. doi: 10.3389/fmicb.2018.01196.
33. Monfredini PM, Souza ACR, Cavalheiro RP, Siqueira RA, Colombo AL. Clinical impact of *Candida* spp. biofilm production in a cohort of patients with candidemia. *Med Mycol* 2018;**56**(7):803-8. doi: 10.1093/mmy/myx133.
34. Lin PY, Chen HL, Huang CT, Su LH, Chiu CH. Biofilm production, use of intravascular indwelling catheters and inappropriate antimicrobial therapy as predictors of fatality in *Chryseobacterium meningosepticum* bacteraemia. *Int J Antimicrob Agents* 2010;**36**(5):436-40. doi: 10.1016/j.ijantimicag.2010.06.033.
35. Muñoz P, Agnelli C, Guinea J, Vena A, Álvarez-Uría A, Marcos-Zambrano LJ, et al. Is biofilm production a prognostic marker in adults with candidaemia? *Clin Microbiol Infect* 2018;**24**(9):1010-5. doi: 10.1016/j.cmi.2018.01.022.
36. Soldini S, Posteraro B, Vella A, De Carolis E, Borghi E, Falleni M, et al. Microbiologic and clinical characteristics of biofilm-forming *Candida parapsilosis* isolates associated with fungaemia and their impact on mortality. *Clin Microbiol Infect* 2018;**24**(7):771-7. doi: 10.1016/j.cmi.2017.11.005.
37. Tumbarello M, Fiori B, Trecarichi EM, Posteraro P, Losito AR, De Luca A, et al. Risk factors and outcomes of candidemia caused by biofilm-forming isolates in a tertiary care hospital. *PLoS One* 2012;**7**(3):e33705. doi: 10.1371/journal.pone.0033705.

38. Tumbarello M, Posteraro B, Trecarichi EM, Fiori B, Rossi M, Porta R, et al. Biofilm production by *Candida* species and inadequate antifungal therapy as predictors of mortality for patients with candidemia. *J Clin Microbiol* 2007;**45**(6):1843-50. doi: 10.1128/jcm.00131-07.
39. Vitális E, Nagy F, Tóth Z, Forgács L, Bozó A, Kardos G, et al. *Candida* biofilm production is associated with higher mortality in patients with candidaemia. *Mycoses* 2020;**63**(4):352-60. doi: 10.1111/myc.13049.
40. Akhter J, Ahmed S, Saleh AA, Anwar S. Antimicrobial resistance and in vitro biofilm-forming ability of *Enterococci* spp. isolated from urinary tract infection in a tertiary care hospital in Dhaka. *Bangladesh Med Res Counc Bull* 2014;**40**(1):6-9. doi: 10.3329/bmrcb.v40i1.20320.
41. Bakhtiari N, Javadmakoei S. Survey on biofilm production and presence of attachment factors in human uropathogenic strains of *Escherichia coli*. *Jundishapur Journal of Microbiology* 2017;**In Press**. doi: 10.5812/jjm-13108.
42. Boroumand M, Sharifi A, Manzouri L, Khoramrooz SS, Khosravani A. Evaluation of *pap* and *sfa* genes relative frequency P and S fimbriae encoding of uropathogenic *Escherichia coli* isolated from hospitals and medical laboratories; Yasuj City, Southwest Iran. *Iranian Red Crescent Medical Journal* 2019;**In Press**. doi: 10.5812/ircmj.89499.
43. Davari Abad E, Khameneh A, Vahedi L. Identification phenotypic and genotypic characterization of biofilm formation in *Escherichia coli* isolated from urinary tract infections and their antibiotics resistance. *BMC Research Notes* 2019;**12**(1):796. doi: 10.1186/s13104-019-4825-8.
44. De Souza GM, Neto E, da Silva AM, Iacia M, Rodrigues MVP, Cataneli Pereira V, et al. Comparative study of genetic diversity, virulence genotype, biofilm formation and antimicrobial resistance of uropathogenic *Escherichia coli* (UPEC) isolated from nosocomial and community acquired urinary tract infections. *Infect Drug Resist* 2019;**12**:3595-606. doi: 10.2147/idr.S228612.
45. Dworniczek E, Piwowarczyk J, Seniuk A, Gościniak G. *Enterococcus*--virulence and susceptibility to photodynamic therapy of clinical isolates from Lower Silesia, Poland. *Scand J Infect Dis* 2014;**46**(12):846-53. doi: 10.3109/00365548.2014.952244.
46. Gad GF, El-Feky MA, El-Rehewy MS, Hassan MA, Abolella H, El-Baky RM. Detection of *icaA*, *icaD* genes and biofilm production by *Staphylococcus aureus* and *Staphylococcus epidermidis* isolated from urinary tract catheterized patients. *J Infect Dev Ctries* 2009;**3**(5):342-51. doi: 10.3855/jidc.241.
47. Garg S, Mohan B, Taneja N. Biofilm formation capability of *enterococcal* strains causing urinary tract infection vis-a-vis colonisation and correlation with *enterococcal* surface protein gene. *Indian Journal of Medical Microbiology* 2017;**35**:48. doi: 10.4103/ijmm.IJMM_16_102.
48. Gawad W, Helmy O, Tawakkol W, Hashem A. Antimicrobial resistance, biofilm formation, and phylogenetic grouping of uropathogenic *Escherichia coli* isolates in Egypt: the role of efflux pump-mediated resistance. *Jundishapur Journal of Microbiology* 2018;**In Press**. doi: 10.5812/jjm.14444.
49. Hashemizadeh Z, Kalantar-Neyestanaki D, Mansouri S. Association between virulence profile, biofilm formation and phylogenetic groups of *Escherichia coli* causing urinary tract infection and the commensal gut microbiota: a comparative analysis. *Microb Pathog* 2017;**110**:540-5. doi: 10.1016/j.micpath.2017.07.046.
50. Kadry AA, Al-Kashef NM, El-Ganiny AM. Distribution of genes encoding adhesins and biofilm formation capacity among uropathogenic *Escherichia coli* isolates in relation to the antimicrobial resistance. *African Health Sciences* 2020;**20**(1):238-47. doi: 10.4314/ahs.v20i1.29.
51. Kudinha T, Johnson JR, Andrew SD, Kong F, Anderson P, Gilbert GL. *Escherichia coli* sequence type 131 as a prominent cause of antibiotic resistance among urinary *Escherichia coli* isolates from reproductive-age women. *J Clin Microbiol* 2013;**51**(10):3270-6. doi: 10.1128/jcm.01315-13.
52. Pullanhi U, Khan S, Vinod V, Mohan K, Kumar A. Outcome of acute urinary tract infections caused by uropathogenic *Escherichia coli* with phenotypically demonstrable virulence factors. *Annals of African medicine* 2019;**18**(3):138-42. doi: 10.4103/aam.aam_49_18.
53. Rahimi F, Katouli M, Karimi S. Biofilm production among methicillin resistant *Staphylococcus aureus* strains isolated from catheterized patients with urinary tract infection. *Microb Pathog* 2016;**98**:69-76. doi: 10.1016/j.micpath.2016.06.031.
54. Shikh-Bardsiri H, Shakibaie MR. Antibiotic resistance pattern among biofilm producing and non producing *Proteus* strains isolated from hospitalized patients; matter of hospital hygiene and antimicrobial stewardship. *Pak J Biol Sci* 2013;**16**(22):1496-502. doi: 10.3923/pjbs.2013.1496.1502.
55. Vuotto C, Grosso F, Longo F, Balice MP, de Barros MC, Peixe L, et al. Biofilm-forming ability and clonality in *Acinetobacter baumannii* strains isolated from urine samples and urinary catheters in different European hospitals. *Adv Exp Med Biol* 2018;**1057**:73-83. doi: 10.1007/5584_2017_70.
56. Alves MJ, Barreira JCM, Carvalho I, Trinta L, Perreira L, Ferreira I, et al. Propensity for biofilm formation by clinical isolates from urinary tract infections: developing a multifactorial predictive model to improve antibiotherapy. *J Med Microbiol* 2014;**63**(Pt 3):471-7. doi: 10.1099/jmm.0.071746-0.
57. Karigoudar RM, Karigoudar MH, Wavare SM, Mangalgi SS. Detection of biofilm among uropathogenic *Escherichia coli* and its correlation with antibiotic resistance pattern. *J Lab Physicians* 2019;**11**(1):17-22. doi: 10.4103/JLP.JLP_98_18.
58. Khodadadian R, Rahdar HA, Javadi A, Safari M, Khorshidi A. Detection of VIM-1 and IMP-1 genes in *Klebsiella pneumoniae* and relationship with biofilm formation. *Microb Pathog* 2018;**115**:25-30. doi: 10.1016/j.micpath.2017.12.036.
59. Neupane S, Pant ND, Khatiwada S, Chaudhary R, Banjara MR. Correlation between biofilm formation and resistance toward different commonly used antibiotics along with extended spectrum beta lactamase production in uropathogenic *Escherichia coli* isolated from the patients suspected of urinary tract infections visiting Shree Birendra Hospital, Chhauni, Kathmandu, Nepal. *Antimicrob Resist Infect Control* 2016;**5**:5. doi: 10.1186/s13756-016-0104-9.

60. Raya S, Belbase A, Dhakal L, Govinda Prajapati K, Baidya R, Kishor Bimali N. *In-vitro* biofilm formation and antimicrobial resistance of *Escherichia coli* in diabetic and nondiabetic patients. *Biomed Res Int* 2019;**2019**:1474578. doi: 10.1155/2019/1474578.
61. Shahbazi S, Asadi Karam MR, Habibi M, Talebi A, Bouzari S. Distribution of extended-spectrum β-lactam, quinolone and carbapenem resistance genes, and genetic diversity among uropathogenic *Escherichia coli* isolates in Tehran, Iran. *J Glob Antimicrob Resist* 2018;**14**:118-25. doi: 10.1016/j.jgar.2018.03.006.
62. Shrestha D, Thapa P, Bhandari D, Parajuli H, Chaudhary P, Thapa K, et al. Biofilm production and antimicrobial resistance among uropathogens in pediatric cases: a hospital based study. *J Nepal Health Res Counc* 2018;**16**(2):178-83. doi: 10.33314/jnhrc.v16i2.921.
63. Shrestha R, Khanal S, Poudel P, Khadayat K, Ghaju S, Bhandari A, et al. Extended spectrum β-lactamase producing uropathogenic *Escherichia coli* and the correlation of biofilm with antibiotics resistance in Nepal. *Annals of Clinical Microbiology and Antimicrobials* 2019;**18**(1):42. doi: 10.1186/s12941-019-0340-y.
64. Bardoloi V, Yogeesha Babu KV. Comparative study of isolates from community-acquired and catheter-associated urinary tract infections with reference to biofilm-producing property, antibiotic sensitivity and multi-drug resistance. *J Med Microbiol* 2017;**66**(7):927-36. doi: 10.1099/jmm.0.000525.
65. Shrestha LB, Baral R, Khanal B. Comparative study of antimicrobial resistance and biofilm formation among Gram-positive uropathogens isolated from community-acquired urinary tract infections and catheter-associated urinary tract infections. *Infect Drug Resist* 2019;**12**:957-63. doi: 10.2147/idr.S200988.
66. Watts RE, Hancock V, Ong CL, Vejborg RM, Mabbett AN, Totsika M, et al. *Escherichia coli* isolates causing asymptomatic bacteriuria in catheterized and noncatheterized individuals possess similar virulence properties. *J Clin Microbiol* 2010;**48**(7):2449-58. doi: 10.1128/jcm.01611-09.
